# Wnt activation as a therapeutic strategy in medulloblastoma

**DOI:** 10.1038/s41467-020-17953-4

**Published:** 2020-08-28

**Authors:** Branavan Manoranjan, Chitra Venugopal, David Bakhshinyan, Ashley A. Adile, Laura Richards, Michelle M. Kameda-Smith, Owen Whitley, Anna Dvorkin-Gheva, Minomi Subapanditha, Neil Savage, Nazanin Tatari, Dillon McKenna, Blessing Bassey-Archibong, Neil Winegarden, Robin Hallett, John P. Provias, Blake Yarascavitch, Olufemi Ajani, Adam Fleming, Gary D. Bader, Trevor J. Pugh, Bradley W. Doble, Sheila K. Singh

**Affiliations:** 1 Section of Neurosurgery, Department of Clinical Neurosciences, University of Calgary, Calgary, AB T2N 1N4 Canada; 2 McMaster Stem Cell and Cancer Research Institute, McMaster University, Hamilton, ON L8S 4K1 Canada; 3 Department of Biochemistry and Biomedical Sciences, Faculty of Health Sciences, McMaster University, Hamilton, ON L8N 3Z5 Canada; 4 Department of Surgery, Faculty of Health Sciences, McMaster University, Hamilton, ON L8N 3Z5 Canada; 5 Princess Margaret Cancer Centre, University Health Network, Toronto, ON M5G 2M9 Canada; 6 Department of Medical Biophysics, University of Toronto, Toronto, ON M5S 1A8 Canada; 7 Department of Molecular Genetics, University of Toronto, Toronto, ON M5S 1A8 Canada; 8 Department of Computer Science, University of Toronto, Toronto, ON M5S 1A8 Canada; 9 Department of Pathology, Faculty of Health Sciences, McMaster University, Hamilton, ON L8N 3Z5 Canada; 10 Northern Biologics, Toronto, ON M5G 1L7 Canada; 11 Department of Pediatrics, Faculty of Health Sciences, McMaster University, Hamilton, ON L8N 3Z5 Canada; 12 The Donnelly Centre, University of Toronto, Toronto, ON M5S 3E1 Canada; 13 Department of Pediatrics and Child Health, University of Manitoba, Winnipeg, MB R3E 0W2 Canada; 14 Department of Biochemistry and Medical Genetics, University of Manitoba, Winnipeg, MB R3E 0W2 Canada

**Keywords:** Cancer therapy, CNS cancer, Cancer stem cells, Paediatric cancer

## Abstract

Medulloblastoma (MB) is defined by four molecular subgroups (Wnt, Shh, Group 3, Group 4) with Wnt MB having the most favorable prognosis. Since prior reports have illustrated the antitumorigenic role of Wnt activation in Shh MB, we aimed to assess the effects of activated canonical Wnt signaling in Group 3 and 4 MBs. By using primary patient-derived MB brain tumor-initiating cell (BTIC) lines, we characterize differences in the tumor-initiating capacity of Wnt, Group 3, and Group 4 MB. With single cell RNA-seq technology, we demonstrate the presence of rare Wnt-active cells in non-Wnt MBs, which functionally retain the impaired tumorigenic potential of Wnt MB. In treating MB xenografts with a Wnt agonist, we provide a rational therapeutic option in which the protective effects of Wnt-driven MBs may be augmented in Group 3 and 4 MB and thereby support emerging data for a context-dependent tumor suppressive role for Wnt/β-catenin signaling.

## Abstract (web summary)

The Wnt molecular subgroup of medulloblastoma is associated with better prognosis than the other molecular subgroups. Here, the authors show that activating Wnt signaling impairs tumor development and improves survival in Group 3 and Group 4 medulloblastoma preclinical models.

## Introduction

Medulloblastoma (MB), the most common malignant pediatric brain tumor, is defined by four molecular subgroups (Wnt, Shh, Group 3, Group 4) based on transcriptional and epigenetic profiles^1,2^. Wnt MB accounts for 10% of cases with the majority harboring somatic *CTNNB1* mutations and chromosomal alterations for monosomy 6^1^. Clinically, Wnt MBs have the most favorable prognosis with a > 95% 5-year survivorship^2^. By contrast, non-Wnt MBs are characterized by metastatic disease, increased rates of recurrence, and intermediate-poor overall survivorship^2^. Given that Wnt MBs represent the only subgroup in which metastasis is not indicative of a poor prognosis^3^, it has been suggested that Wnt signaling may contribute to their remarkable response to therapy^4–8^. Since prior reports have illustrated the antitumorigenic role of Wnt activation in sonic hedgehog (Shh)-driven MB^8^, this work primarily focuses on Group 3 and 4 MB, while extending some findings to Shh MB. However, unlike Group 3 and 4 MBs, which are Wnt-naive tumors, Shh MBs harbor baseline Wnt activation^9^, which may suggest their dependence on Wnt signaling for tumorigenesis and thereby confound the therapeutic effects of Wnt activation. Herein, using primary patient-derived MB brain tumor-initiating cell (BTIC) lines, we characterize intrinsic differences in the tumor-initiating capacity of Wnt, Group 3, and Group 4 MBs. We further describe the impaired tumorigenic potential of endogenous Wnt-active cells isolated from non-Wnt MBs. By treating MB xenografts with a substrate-competitive peptide Wnt agonist, we show Wnt activation to serve as a rational therapeutic option. Specifically, our preclinical work provides evidence for the context-specific tumor suppressive function of the Wnt/β-catenin pathway and establishes activated Wnt signaling as a mechanism for potentially targeting Group 3 and 4 MB.

## Results

### MB BTICs retain subgroup affiliation

To assess the biological validity of our MB BTIC model, we asked if gene expression differences between subgroups in bulk MB manifested themselves in our model. We performed differential expression analysis from bulk MB data^10^ to identify upregulated genes specific to each subgroup, and scored both the bulk MB data and our MB BTIC lines for these upregulated gene expression signatures using single-sample gene set enrichment analysis (ssGSEA)^11^. As expected, upregulated genes associated with Wnt (*n* = 6326), Group 3 (*n* = 7491), and Group 4 (*n* = 6510) showed strongest positive enrichment in bulk Wnt, Group 3, and Group 4 MB samples, respectively, validating the specificity of the signatures (Supplementary Fig. 1a). Interestingly, the Wnt, Group 3, and Group 4 MB signatures showed strongest relative enrichment in our Wnt (BT853), Group 3 (SU_MB002), and Group 4 (ICB1299) MB BTIC lines, respectively (Supplementary Fig. 1b), showing preservation of subgroup affiliation among our MB BTIC lines.

Using principal component analyses (PCA), we examined the inter-subgroup heterogeneity between MB BTIC lines from Wnt, Group 3, and Group 4 MBs (Supplementary Fig. 2a). RNA-seq data from these lines yielded distinct gene expression profiles between our Wnt, Group 3 and, Group 4 MB lines (Supplementary Fig. 2b–e, Supplementary Data 1). Specifically, several genes implicated in the self-renewal of malignant stem cell populations and previously described by our group to be enriched in poor-outcome MBs^12^ were more highly expressed in Group 3 and 4 MBs when compared with Wnt MB (Supplementary Fig. 2f). Bmi1 and Sox2 are hallmark master regulatory stem cell genes essential to BTIC self-renewal, and thus enriched in experimental BTIC models^13–15^. Gene set enrichment analysis (GSEA) of non-Wnt MB (Group 3 MB line (Supplementary Fig. 3a) and Group 4 MB line (Supplementary Fig. 3b)) showed enrichment of genes in the *Bmi1* signature, which function as key epigenetic regulators of fate determination and self-renewal in normal and malignant cerebellar stem cells^14^. By contrast, cell cycle checkpoint and apoptosis gene signatures were more active in Wnt MB lines (Supplementary Fig. 3b) when compared with Group 4 MBs. No differences were identified in cell cycle checkpoint and apoptosis gene signatures between Wnt and Group 4 MB lines. Pathway network analysis further identified an increase in DNA replication, transcriptional regulation, ribosomal processing, and translational regulation in Group 3 and 4 MB lines (Supplementary Fig. 3c, Supplementary Data 2), suggestive of a hyperproliferative state.

Additional differences between Wnt, Group 3, and Group 4 MBs were determined using in vitro and in vivo tumorigenic assays. TCF reporter assays showed a significant increase in endogenous Wnt activity in Wnt compared with Group 3 and 4 MB lines (Supplementary Fig. 3d). Similar to the Wnt-mediated inhibition of cerebellar stem cell self-renewal^16^, proliferation (Supplementary Fig. 4a–b) and self-renewal (Supplementary Fig. 4c–d) were impaired in our Wnt MB line (BT853) when compared with Shh (Daoy), Group 3 (SU_MB002, HD-MB03, D425, D458), and Group 4 (ICB1299) MB lines. It should be noted that the Daoy cell line contains a homozygous TP53 deletion, which is not seen in Shh MB, and results must therefore be reviewed in context for data with the Daoy cell line. Xenografts generated with 5.0 × 10^5^ Wnt MB cells led to tumor formation in 4/5 mice, whereas no tumors formed with 1.0 × 10^5^ cells (0/5 mice). By contrast, all Group 3 MB xenografts formed tumors at both numbers (10/10 mice) (Supplementary Fig. 4e). Overall tumor volume was higher in Group 3 than Wnt MB xenografts (Supplementary Fig. 4f). Wnt MBs also contained a marked survival advantage compared with Group 3 MBs for xenografts generated from both 1.0 × 10^5^ (Supplementary Fig. 4g) and 5.0 × 10^5^ cells (Supplementary Fig. 4h). The observed in vivo growth differences are most likely reflective of the in vitro differences in proliferation and self-renewal between Wnt and Group 3 MBs. These findings support the clinical observations of prognostic variations among MB subgroups.

### Ectopic Wnt activation in MB BTICs impairs tumorigenesis

Previous work hypothesized the improved outcome in Wnt MB to be due to the secretion of soluble Wnt antagonists by tumor cells that may impair the blood–brain barrier, rendering it more susceptible to chemotherapy^7^. Our data suggests the inhibition of self-renewal pathways to explain the improved outcome seen in Wnt MB. The integrity of the Wnt/β-catenin pathway in Group 3 MBs was assessed using Wnt3A-conditioned medium. A significant increase in *Axin2* expression, a conserved downstream Wnt target gene^17^, was noted in SU_MB002 cells cultured in Wnt3A-conditioned medium (Supplementary Fig. 5a). Wnt3A-mediated pathway activation also reduced secondary tumor sphere size (Supplementary Fig. 5b–c), frequency (Supplementary Fig. 5d), and cell proliferation in non-Wnt MBs (Supplementary Fig. 5e). These findings support a transient alteration in the oncogenic phenotype of aggressive MBs following Wnt activation.

**Fig. 1:**
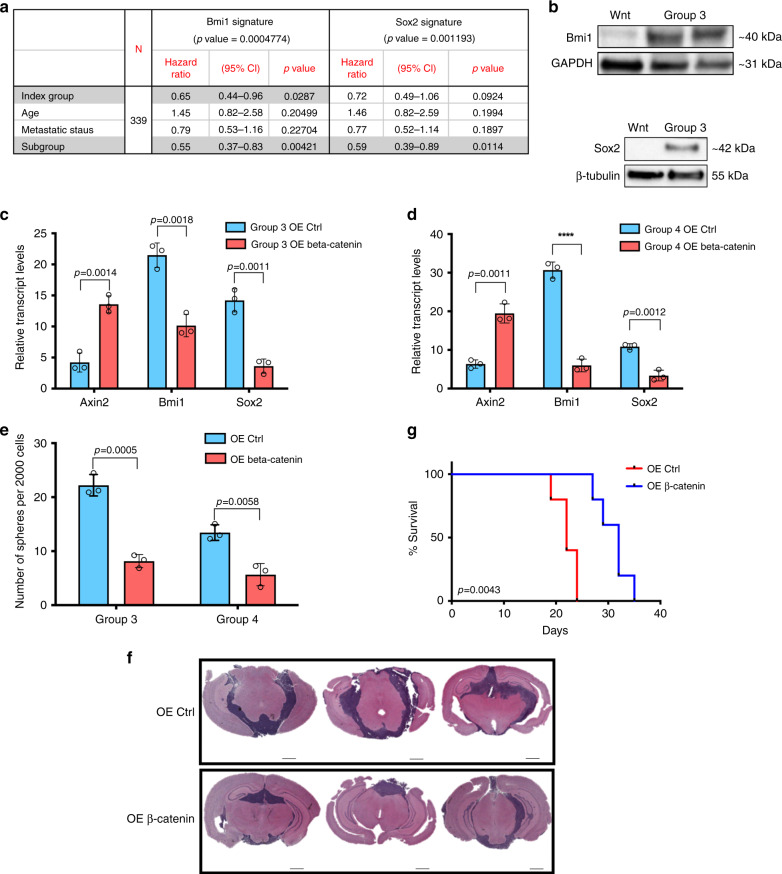
Ectopic expression of β-catenin in treatment-refractory MBs reduces self-renewal gene expression and tumor burden while increasing overall survival. **a** Multivariate survival analysis including index, age, metastatic status, and subgroup shows significant association with patient survival using the *Bmi1* (*p* = 0.0004774) and *Sox2* (*p* = 0.001193) signatures. **b** Bmi1 and Sox2 protein levels are significantly reduced in Group 3 (HD-MB03, D425) MB lines when compared with Wnt MB (BT853) (*n* = 3, independent experiments per MB line, 1 line per subgroup). Differential *Axin2* (Group 3: *p* = 0.0014, Group 4: *p* = 0.0011), *Bmi1* (Group 3: *p* = 0.0018, Group 4: *p* = 0.000087), and *Sox2* (Group 3: *p* = 0.0011, Group 4: *p* = 0012) transcript levels following β-catenin overexpression in (**c**) Group 3 and (**d**) Group 4 MB lines (*n* = 3, independent experiments per MB line, 1 line per subgroup, all samples normalized to *GAPDH*). **e** Tumor sphere formation is impaired following β-catenin overexpression in Group 3 (*p* = 0.0005) and Group 4 MBs (*p* = 0.0058) (*n* = 3, independent experiments per MB line, 1 line per subgroup). **f** Representative histology images showing significant reduction in tumor formation in xenografts generated from Group 3 β-catenin-overexpressing lines (*n* = 5) compared with control (*n* = 5). **g** β-catenin overexpression xenografts (*n* = 5, median survival 32.0 days) display a significant increase in overall survival when compared with control xenografts (*n* = 5, median survival 22.0 days) (*p* = 0.0043). Histology image scale bar = 5000 μm. Panels (**c**–**e**) contain error bars expressed as mean ± standard error (mean) using two-tailed, unpaired Student’s *t* test. Panel (**g**) analyzed using log-rank (Mantel–Cox) test. ****p < 0.0001.

Since developmental processes may be carried forward from ontogeny into oncology and both Bmi1 and Sox2 have been implicated in cerebellar and MB development^14,18^ and specifically as surrogates for treatment-refractory BTICs^18,19^, we investigated their clinical utility in a cohort of 377 Group 3 and 4 MBs^10^. Using a *Bmi1* gene signature that predicts metastasis, tumor progression, and death from cancer^20^, we found a reduced overall survivorship for patients with elevated expression of the signature (Supplementary Fig. 6a). Using a similar approach, a *Sox2* gene signature^21^ identified *Sox2*^high^ patients to have a significant reduction in overall survival (Supplementary Fig. 6b). To further understand factors associated with patient outcome, we performed univariate and multivariate survival analyses, which included the index, age, metastatic status, and subgroup. *Bmi1* signature genes (Fig. 1a, Supplementary Table 1) showed a significant association with patient outcome in a univariate analysis (*p* = 0.0113), which remained significant (*p* = 0.0287) when adjusted for age, metastatic status, and subgroup. Metastatic status alone was not significantly associated with patient outcome in both univariate and multivariate analyses, but it was significantly associated with the index (*p* = 0.03339). The *Sox2* signature index (Fig. 1a, Supplementary Table 2) showed a significant association with patient survival in univariate analysis (*p* = 0.00441) but became a trend when adjusted for age, metastatic status, and subgroup for the multivariate analysis (*p* = 0.0924). An association was noted between metastatic status and subgroup affiliation (*p* = 0.03888, *p* = 0.02943 respectively). The expression of both self-renewal genes *Bmi1* and *Sox2* were reduced in Wnt MB BTICs (BT853) when compared with Shh (Daoy), Group 3 (SU_MB002, HD-MB03, RCMB40, D425, Med8A, D458) and Group 4 (ICB1299) MB BTICs (Fig. 1b and Supplementary Figs. 7 and 8), indicating differences in self-renewal activity across MB subgroups.

Considering that *CTNNB1*, which encodes β-catenin, is mutated in 86% of Wnt MBs, we ectopically expressed a stabilized β-catenin mutant in Group 3 and 4 MB lines. *Axin2* expression was used to determine sufficient overexpression (Fig. 1c, d). *Bmi1* and *Sox2* were reduced following β-catenin overexpression (Fig. 1c, d). The self-renewal capacity of β-catenin-overexpressing lines was markedly reduced when compared with controls (Fig. 1e); it should be noted that no direct comparison was made to native Wnt models in these experiments. Orthotopic injections of Group 3 MB cells revealed a significant reduction in overall tumor burden (Fig. 1f, Supplementary Fig. 9) and increase in survival (Fig. 1g) following the ectopic expression of β-catenin.

### Non-Wnt MBs contain Wnt-active cells that are less tumorigenic

**Fig. 2:**
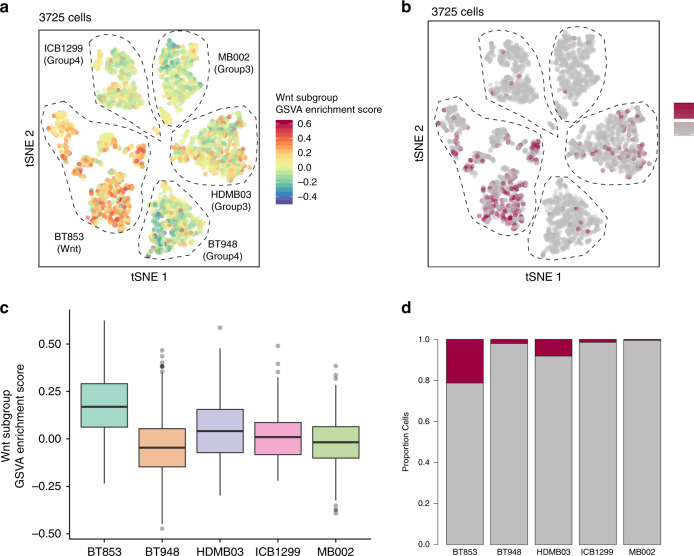
Characterization of Wnt activity in Wnt, Group 3, and Group 4 MBs. Heterogeneous Wnt subgroup scored across patient-derived MB cultures. **a** tSNE of 3725 cells colored by GSVA enrichment score for the Wnt subgroup gene signature. **b** Cells colored by classification for enrichment of Wnt subgroup signature. Dark red cells surpassed the 5% cutoff and are considered significantly enriched for the gene signature. Gray cells did not pass the threshold. **c** Wnt subgroup signature scores for each sample. Each point in the box plot represents a cell (*n* = 3725 cells from five samples). Box plots represent the median, upper, and lower quartiles of the distribution and whiskers represent 1.5x IQR or the most extreme value. Outliers represented as circles. **d** Proportion of cells enriched for the Wnt subgroup gene signature per sample.

**Fig. 3:**
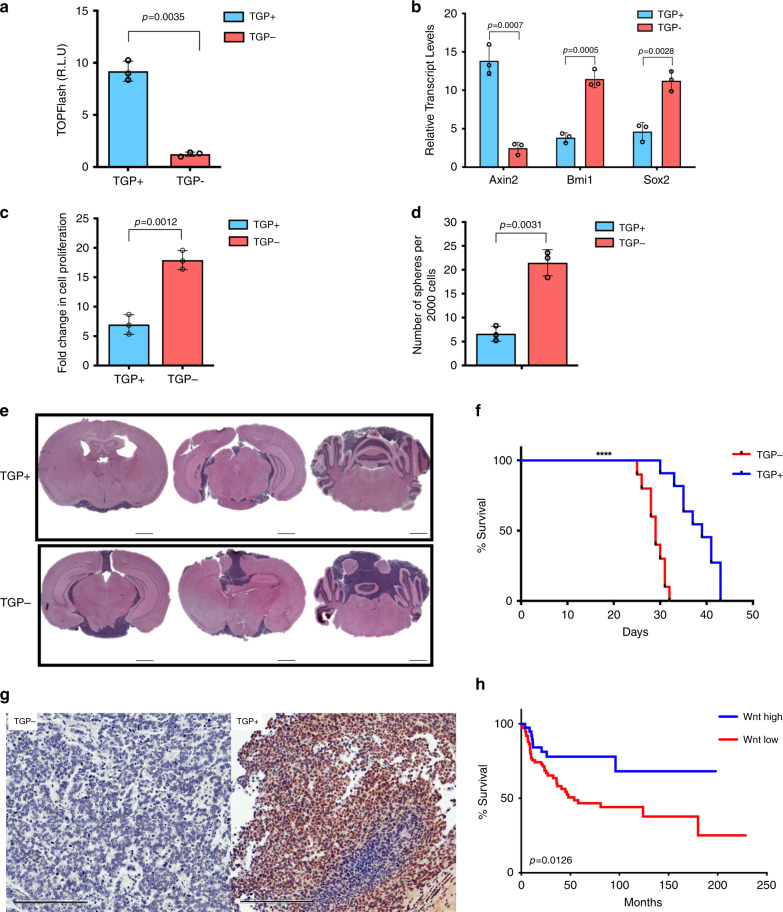
Rare endogenous Wnt-active MB cells from treatment-refractory MBs have reduced tumorigenic properties. **a** SU_MB002 cells transduced with a Wnt reporter show enhanced TCF reporter activity in TGP+ cells compared with TGP− cells (*p* = 0.0035) (*n* = 3, independent experiments). **b** Differential *Axin2* (*p* = 0.0007), *Bmi1* (*p* = 0.0005), and *Sox2* (*p* = 0.0028) transcript levels in TGP+ and TGP− cells (*n* = 3, independent experiments, all samples normalized to *GAPDH*). **c** Proliferative (*p* = 0.0012) and **d** self-renewal capacity (*p* = 0.0031) of TGP+ cells are significantly reduced when compared with TGP− cells (*n* = 3, independent experiments). **e** Representative histology images illustrating reduced tumor burden in TGP+ (*n* = 10) compared with TGP− xenografts (*n* = 10). **f** TGP+ xenografts (*n* = 10, median survival 39.0 days) display a significant increase in overall survival when compared with TGP− xenografts (*n* = 10, median survival 29.0 days) (*p* = 0.000012). **g** TGP+ tumors revert to TGP− as deduced by IHC for GFP expression. **h** Treatment-refractory MB patients (*n* = 113) with high expression of the Wnt hallmark signature have an improved overall survivorship (median survival undetermined) when compared with those patients with low Wnt hallmark signature expression (median survival 54.0 months) (*p* = 0.0126). Xenograft H/E histology image scale bar = 5000 μm. IHC histology image scale bar = 50 μm. Panels (**a**–**d**) contain error bars expressed as mean ± standard error (mean) using two-tailed, unpaired Student’s *t* test. Panels (**f**, **h**) analyzed using log-rank (Mantel–Cox) test. ****p < 0.0001.

While genetic heterogeneity exists within individual MB subgroups^10^, general subgroup affiliation does not change at recurrence^22^, metastasis^23^, or within different regions of a tumor^24^. However, much of this work has been done on bulk tumor samples without considering subclonal variations observed at a single-cell level. Given that emerging data in other malignancies have implicated subclonal genetic drivers in disease progression and relapse^25,26^, we performed single-cell RNA-seq on Wnt, Group 3, and Group 4 MB lines (Fig. 2a, Supplementary Figs. 10–14) to understand the functional relevance of subclonal Wnt activity in MB cells. As expected, cells from the Wnt MB line had the highest enrichment for the Wnt subgroup gene signature (Fig. 2b–d). Intriguingly, we found that a small population of cells in Group 3 and 4 MB lines exhibited high Wnt subgroup signature scores, similar to cells of Wnt subgroup origin (Fig. 2b). These results support the presence of a rare population of cells in Group 3 and 4 MBs that resemble Wnt subgroup cells. We validated our findings in four independent datasets consisting of scRNA-seq^27,28^ (Supplementary Figs. 15 and 16) and bulk tumor RNA-seq data^1,10^ (Supplementary Figs. 17 and 18). We then wondered if these Wnt-active cells maintained the reduced tumorigenic potential observed in Wnt MB. Using a lentiviral Wnt reporter (7XTCF-GFP)^29^, we identified rare Wnt-active cells (TGP+) from Group 3 MB cells (Fig. 3). Both Wnt-active (TGP+) and Wnt-inactive (TGP−) cells were isolated using flow cytometric cell sorting (Supplementary Fig. 19a–b). When TGP+ and TGP− cells were cultured in vitro, we observed TGP+ convert to TGP− cells and vice versa, but the majority of them repopulated to TGP− cells (Supplementary Fig. 19c). Wnt activity in TGP+ cells was validated using the TCF Wnt reporter assay (Fig. 3a) and *Axin2* transcript levels (Fig. 3b). TGP+ cells contained reduced levels of *Bmi1* and *Sox2* transcript (Fig. 3b) and protein expression (data not shown) when compared with TGP− cells. Decreased proliferative (Fig. 3c) and self-renewal (Fig. 3d) indices were also noted in TGP+ compared with TGP− cells. Separate xenografts generated from orthotopic injections of TGP+ or TGP− cells revealed a reduced tumor burden (Fig. 3e, Supplementary Fig. 20a–b) and enhanced survival (Fig. 3f) in TGP+ xenografts compared with TGP−, with some TGP+ cells reverting back to TGP− cells as observed by immunostaining for GFP positivity in these xenografts (Fig. 3g). These data highlight the intrinsic tumor suppressive role of activated Wnt signaling in MB. To examine the clinical utility of our model systems, we used the Wnt hallmark gene signature to probe a clinically annotated dataset of 113 Group 3 human MBs^10^ for differences in overall survival. Group 3 patients with high expression of the Wnt hallmark signature maintained a longer overall survival compared with those with low expression (Fig. 3h). As a result, endogenous Wnt activity in Group 3 MBs appears predict improved survivorship in MBs that are otherwise metastatic and refractory to current treatment. To further understand factors associated with patient outcomes, we performed univariate and multivariate survival analyses, which included the index, age and metastatic status. The index based on the Wnt signature (see Supplementary Table 3) was the only factor that showed a significant association with patient survival in univariate analysis (*p* = 0.0158) and when adjusted for other factors, such as age and metastatic status (*p* = 0.0103).

### Pharmacological Wnt activation in MB xenografts improves survival

To develop a therapeutic strategy employing Wnt activation, we initially used the small molecule GSK-3 inhibitor, CHIR99021, which acts as a competitive inhibitor of ATP binding^30^. GSK-3 functions to inhibit Wnt/β-catenin signaling by phosphorylating the downstream Wnt effector, β-catenin, rendering it nonfunctional. TCF reporter measured Wnt activation following exposure to CHIR99021 in Group 3 and 4 MB lines (Supplementary Fig. 21a–b). *Axin2* transcript levels (Supplementary Fig. 21c–d) and increase in nuclear and cytoplasmic β-catenin levels (Supplementary Fig. 22) provided further validation of Wnt activation. *Bmi1* and *Sox2* expression were decreased following CHIR99021 treatment (Supplementary Fig. 21c–d). Although CHIR99021 also reduced the proliferative and self-renewal abilities (Supplementary Fig. 21e–f) of Group 3 and 4 MB lines, we were unable to proceed to preclinical studies due to limited blood–brain barrier permeability.

**Fig. 4:**
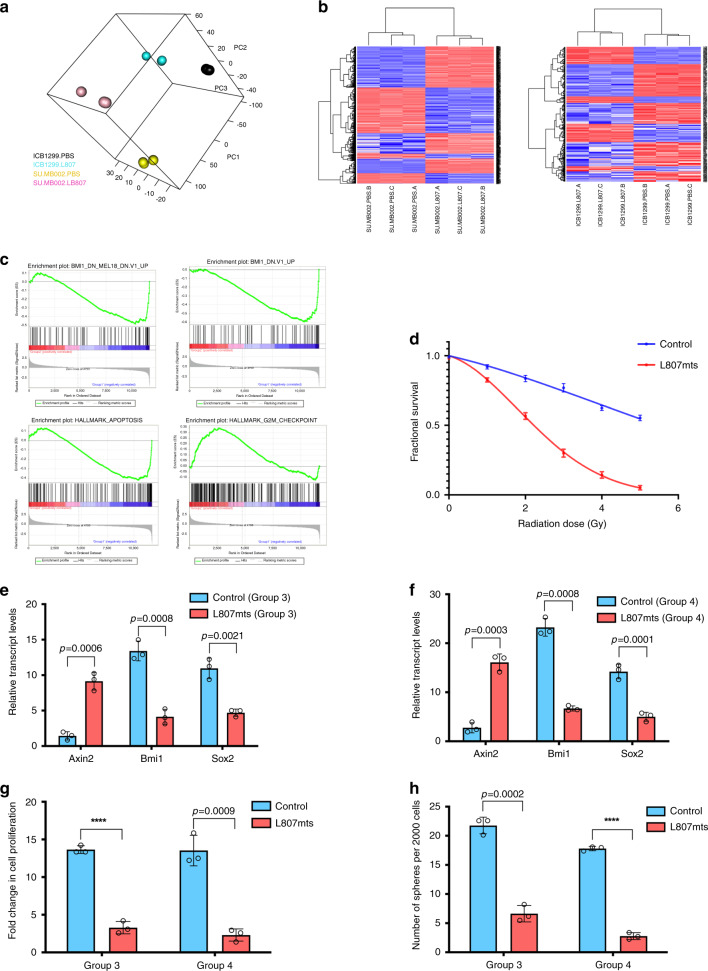
Pharmacological activation of Wnt signaling in MB impairs stem cell properties. **a** Visualization of the first three principal components from PCA of Group 3 and 4 lines treated with small molecule Wnt activator (L807mts) or control (PBS) (*n* = 3, independent samples per MB line, 1 line per subgroup). **b** Heatmaps of differentially expressed genes between L807mts- and PBS-treated Group 3 (SU_MB002, left panel) and Group 4 (ICB1299, right panel) MB lines (*n* = 3, independent samples per MB line, 1 line per subgroup). **c** GSEA enrichment plots showing that Bmi1 associated genes are significantly reduced while apoptosis and cell cycle inhibitors are enriched in L807mts-treated cells compared with control. **d** L807mts-treated cells are much more radiosensitive than control cells (1 Gy *p* = 0.01145; 2 Gy *p* = 0.0013; 3 Gy *p* = 0.0004; 4 Gy *p* = 0.00008; 5 Gy *p* = 0.00004) (*n* = 3, independent experiments). Differential *Axin2* (Group 3: *p* = 0.0006, Group 4: *p* = 0.0003), *Bmi1* (Group 3: *p* = 0.0008, Group 4: *p* = 0.0008), and *Sox2* (Group 3: *p* = 0.0021, Group 4: *p* = 0.0001) transcript levels in L807mts-treated (**e**) Group 3 and (**f**) Group 4 MB lines (*n* = 3, independent experiments per MB line, 1 line per subgroup, all samples normalized to *GAPDH*). Both, **g** proliferation (Group 3: *p* = 0.00005, Group 4: *p* = 0.0009) and **h** self-renewal (Group 3: *p* = 0.0002, Group 4: *p* = 0.000003) are impaired following L807mts treatment in Group 3 and Group 4 MB lines (*n* = 3, independent experiments per MB line, 1 line per subgroup). Panels (**d**–**h**) contain error bars expressed as mean ± standard error (mean) using two-tailed, unpaired Student’s *t* test. ****p < 0.0001.

**Fig. 5:**
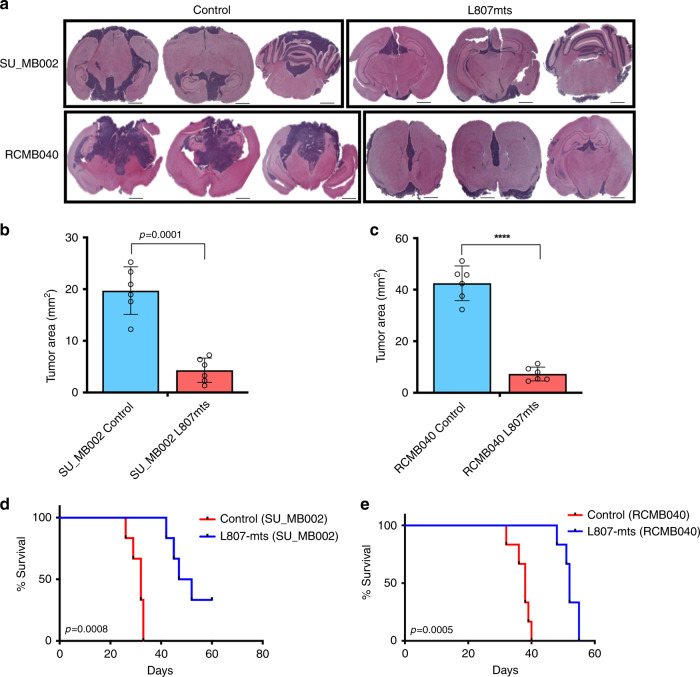
Pharmacological activation of Wnt signaling in MB reduces tumor formation and improves overall survival. **a** Representative histology images of xenografts generated from patient-derived treatment-refractory MBs following L807mts treatment (*n* = 6 per MB line) contain a reduction in overall tumor burden when compared with xenografts treated with PBS control (*n* = 6 per MB line). **b** SU_MB002 (*n* = 12, *p* = 0.0001) **c** RCMB40 (*n* = 12, *p* = 0.000011), treated with L807mts (*n* = 6, individual mice per MB line) contain a significant reduction in overall tumor volume when compared with PBS control-treated xenografts (*n* = 6, individual mice per MB line). Xenografts of **d** SU_MB002 (*n* = 12; median survival L807mts 49.5 days, PBS 32.0 days; *p* = 0.0008, *n* = 12), and **e** RCMB40 (*n* = 12; median survival L807mts 52.9 days, PBS 38.0 days; *p* = 0.0005) treated with L807mts display a significant survival advantage when compared with control PBS-treated mice. Histology image scale bar = 5000 μm. Panels (**b**, **c**) contain error bars expressed as mean ± standard error (mean) using two-tailed, unpaired Student’s *t* test. Panels (**d**, **e**) analyzed using log-rank (Mantel–Cox) test. ****p < 0.0001.

In an attempt to find a suitable preclinical molecule, we identified L807mts, a GSK-3 inhibitor that functions through a substrate-to-inhibitor conversion mechanism within the GSK-3 catalytic site^31^. TCF reporter activity confirmed Wnt activation in L807mts-treated cells (Supplementary Fig. 23a–b), which was further corroborated with *Axin2* expression (Fig. 4e, f) and β-catenin expression (Supplementary Fig. 22). PCA was used to examine changes in the transcriptional machinery following L807mts treatment (Fig. 4a). RNA-seq data from L807mts-treated cells showed diverging gene expression profiles (Fig. 4b, Supplementary Fig. 23c–e, Supplementary Data 2). In order to assess the activation of Wnt by L807mts treatment, we examined enrichment of the top 50 and top 100 signatures in the samples treated with L807mts relative to the matching samples treated with PBS. Enrichment analysis was performed with GSEA^32,33^. Top genes upregulated in Wnt MBs were found to be enriched in corresponding Group 3 and 4 samples treated with L807mts. This provides supportive evidence for the observed activation of Wnt signaling in Group 3 and 4 MBs treated with L807mts (Supplementary Fig. 23f–g). The Bmi1 gene signature was also reduced in L807mts-treated samples as per GSEA (Fig. 4c). Additional GSEA identified an increase in the expression of apoptotic and G2M checkpoint-related gene signatures in L807mts-treated samples (Fig. 4c). Our single-cell RNA-seq data also revealed that several Wnt activation genes that were highly enriched in endogenous single cells from Group 3 and 4 MBs, including NFATC4 and GALNT14, were also upregulated in L807mts-treated Group 3 and 4 MB lines (NFATC4 fc = 4.33, GALNT14 fc = 2.43, Supplementary Data 2). These findings support the activation of Wnt-driven tumor suppressive pathways following L807mts treatment. Given the enhanced expression of apoptotic and cell cycle inhibition pathways in L807mts-treated cells and prior reports that have indicated *TP53*-mutated Wnt MBs to be more radiosensitive than *TP53*-mutated non-Wnt MBs^4,5^, we investigated the potential to radiosensitize treatment-refractory MBs with L807mts. An increase in radiosensitization was observed with L807mts treatment in vitro (Fig. 4d) and in vivo (Supplementary Fig. 24). L807mts treatment also decreased *Bmi1* and *Sox2* (Fig. 4e, f) levels and impaired the proliferation and self-renewal (Fig. 4g, h) of Group 3 and 4 MB lines. Preclinical studies were performed on three separate patient-derived MB lines (Shh: BT992; Group 3: SU_MB002, RCMB040). A reduction in overall tumor burden (Fig. 5a–c, Supplementary Fig. 25a–b) and increase in overall survival (Fig. 5d, e, Supplementary Fig. 25c) were observed in xenografts treated with L807mts. These preclinical studies provide evidence in support of a highly specific small molecule Wnt agonist with potential clinical utility for treating MB.

## Discussion

Current MB clinical trials have based risk-adapted therapy on molecular subgroups. Trials focused on Wnt-driven MB are deescalating chemo/radiotherapy (NCT01878617, NCT02724579, NCT02212574). By contrast, trials for Group 3 and 4 MBs are escalating therapy with the hope of improving survivorship with recurrent MB patients treated with palliation alone^2^. Given the few targeted treatment options for Group 3 and 4 MBs, our work highlights a rational therapeutic option in which the protective effects of Wnt-driven MBs may be augmented in Group 3 and 4 MBs through targeted Wnt activation. While our more limited findings in Shh MB (Supplementary Figs. 4b, 4d, 7, 23) align with prior reports describing an antitumorigenic role for Wnt activation in murine models of Shh MB^8^, the presence of endogenous Wnt activity in Shh MB^9^ may confound the therapeutic effects of Wnt activation and thus requires further investigation. This work illustrates an emerging paradigm in Wnt biology in which functions of the pathway may be conserved in a context-dependent manner^34–39^, such as a hindbrain-specific function in which self-renewal is impaired from ontogeny to oncology. However, before considering Wnt activation as a treatment paradigm in childhood MB, it is important to identify the mechanisms by which Wnt/β-catenin signaling function to target self-renewal genes that could impede growth of these aggressive MBs. As molecular oncology trials continue to develop, approaches to overcome the dependence of tumors on malignant pathways are warranted and as such we provide a therapeutic rationale with clinical appeal that may alter our approach to cancer by reactivating anti-oncogenic programs that have been developmentally silenced in a tissue-specific manner.

## Methods

### Culture of primary MB samples

Primary human pediatric MBs, BT853, and BT992 were obtained from consenting patients and families as approved by the Hamilton Health Sciences/McMaster Health Sciences Research Ethics Board. BT853 is a primary Wnt subgroup MB, whereas BT992 is a primary Shh subgroup from a patient who later relapsed. Both samples were dissociated in PBS containing 0.2 Wünsch unit/mL of Liberase Blendzyme 3 (Roche), and incubated at 37 °C in a shaker for 15 min. The dissociated tissue was filtered through a 70 μm cell strainer and collected by centrifugation (450 *g*, 3 min). Tumor cells were resuspended in a serum-free BTIC enrichment medium, and replated on ultra-low attachment plates (Corning). Additional primary human pediatric MB cultures were obtained from collaborators as kind gifts. SU_MB002, a treatment-refractory Group 3 MB acquired at autopsy was received from Dr. Yoon-Jae Cho. Dr. Robert Wechsler-Reya provided RCMB40. Dr. Till Milde provided HD-MB03, a treatment-naive Group 3 MBs. ICB1299, a treatment-naive Group 4 MB was obtained from Dr. Silvia Marino. D425, D458 (Group 3) and Daoy (Shh subgroup) were commercially available cell lines. All samples were cultured in BTIC enrichment medium for at least 48 h prior to experimentation. BTIC enrichment medium was composed of NeuroCult complete medium (STEMCell Technologies, 10 ng/mL bFGF, 20 ng/mL EGF, 2 μg/mL heparin). Expansion medium was used prior to experimentation and BTIC enrichment. SU_MB002 was expanded using the same BTIC enrichment medium BT853, BT992, and RCMB40 were expanded with the BTIC enrichment medium supplemented with 10% fetal bovine serum (FBS). ICB1299 was expanded with Dulbecco’s Modified Eagle’s Medium high glucose (Life Technologies #11965-118) supplemented with 10% FBS.

### RNA-seq

Total RNA was extracted using the Norgen Total RNA isolation kit and quantified using a NanoDrop Spectrophotometer ND-1000. The RNA was sequenced using single-end 50 bp reads on the Illumina HiSeq platform (Illumina, San Diego CA, USA). Raw sequence data were exported to FASTQ format and were filtered based on quality scores (Quality cutoff of 20 for at least 90% of the bases in the sequence). Next the reads were mapped to the UCSC mRNA transcript human database based on the GRCh38/hg38 version using HISAT^40^. The counts were obtained by using ht-seq count with the “intersection-strict” option^40^. Counts were transformed with TMM transformation and then normalized with VOOM (package “limma” in R)^41^. Distributions of samples were examined by performing PCA with “rgl” package in R (https://cran.r-project.org/web/packages/rgl/index.html) and by examining a dendrogram built with “hclust” function from “stats” package in R with Euclidean distance and average linkage.

Differential expression was obtained by using “limma” package, *p* values were adjusted for multiple testing with BH method^42^ and resulting values <0.05 were considered to be significant. Pathway analysis was performed by using the Reactome tool (https://reactome.org/PathwayBrowser/#TOOL = AT), and only terms yielding FDR < 0.05 were considered to be significant and were used for further examination.

GSEA^32,33^ was performed by using Oncogenic (C6) and Hallmark MSigDB collections of gene sets. Further GSEA analysis was performed using top 50 and top 100 genes belonging to the signatures obtained from the differential expression analysis.

Survival analysis was performed by using the following signatures: (1) Sox2 signature (BENPORATH_SOX2_TARGETS signature deposited in C2 MSigDB collection), (2) WNT Hallmark signature (HALLMARK_WNT_BETA_CATENIN_SIGNALING signature deposited in the Hallmark MSigDB collection), and (3) BMI1 Glinsky signature^20^. For each signature a signature score was calculated for each patient: $\frac {\sum _{1LR} {x}_{1}} {n_{R}}$, where *x* is the log2-transformed expression, *R* is the set of genes comprising the signature of interest, and *n* is the number of genes in that signature, similar to as described previously^43,44^ but using only the genes regulated in the same direction. For each signature, patients were divided into “High” and “Low” signature index groups using tertiles (1/3 of the patients were assigned to the “High” category and 2/3 were assigned to the “Low”category). Additional factors, such as age, metastatic status, and subgroup were used. Univariate and multivariate survival analyses were performed using the “survival” package in R (https://cran.r-project.org/web/packages/survival/index.html).

### Comparative analysis of transcriptomic data of bulk MB and MB BTIC lines

All analyses were performed in R 3.4.4. The RNA microarray data was downloaded from GEO (GSE85217)^10^. Before performing analyses with this data, the data were subsetted for 540 WNT, Group 3 and Group 4 samples (i.e. no SHH). In addition, ENSG identifiers were mapped to HGNC symbols using Ensembl version 77, with mappings downloaded using the BioMART package^45,46^.

Differential expression was conducted using voom and limma for RNA-seq data (this study) and using limma for microarray data (Cavalli 2017 data). Differentially expressed genes were filtered at false discovery rate (FDR) 0.05 (Benjamini–Hochberg procedure). Differential expression was performed for the Cavalli data in three ‘one vs. all others’ comparisons (one for each MB subtype), and differential expression was calculated in a similar manner for untreated MB stem cell lines from this study.

The Cavalli et al. dataset^10^ and our MB BTIC samples were scored for gene expression signatures separately using ssgsea^11^ as implemented in the GSVA package^47^, using default settings for GSVA::gsva(expr = expression.matrix, gset.idx.list = gene sets, method = ‘ssgsea’), where expression.matrix is an expression matrix, gene sets is a list of gene sets, and method ‘ssgsea’ specifies to use ssgsea method for scoring.

### Wnt/TCF reporter assay

MB cells were cotransfected with the constructs 8XTOPFlash (1.8 mg), driving firefly luciferase, and pRL-CMV (0.2 mg), driving the expression of Renilla luciferase for normalization (Promega). After 24 h, MB cells were supplemented with BTIC enrichment medium. Cells were washed twice with PBS 24 h following medium change and were lysed with passive lysis buffer (Promega). The luciferase reporter activities were measured using a luminometer as per the manufacturer’s instructions (Promega Dual-Light System).

### Cell proliferation assay

Single cells were plated in 96-well plates, at a density of 1000 cells/200 μL per well in quadruplicate for each sample and incubated for four days. Twenty microliters of Presto Blue (Life Technologies), a fluorescent cell metabolism indicator, was added to each well ~4 h prior to the readout time point. Fluorescence was measured with a FLUOstar Omega Fluorescence 556 Microplate reader (BMG LABTECH) at an excitation and emission wavelength of 540 and 570 nm, respectively. Readings were analyzed using the Omega software.

### Secondary sphere formation analysis

SU_MB002 MB tumorspheres were mechanically dissociated with a 1000 μL pipette tip. Cells were plated at 200 cells per well in 200 μL of BTIC enrichment medium in a 96-well plate. Cultures were left undisturbed at 37 °C with 5% CO_2_. After 3 days, the number of secondary spheres per well was counted and used to estimate the mean number of spheres/2000 cells.

### In vivo experiments

All in vivo studies were performed according to McMaster University Animal Research Ethics Board approved protocols. Intracranial injections were performed as previously described^48^ using the following human MB samples: BT853, BT992, SU_MB002, RCMB40. Briefly, the appropriate number of live cells, determined by trypan blue exclusion, was resuspended in 10 μL of PBS. NOD-SCID mice were anaesthetized using isofluorane gas (5% induction, 2.5% maintenance) and cells were injected into the frontal lobe using a 10 μL Hamilton syringe.

With regards to specific treatment groups and injected cell numbers, comparative tumor-initiating capacities of BT853 and SU_MB002 were performed at limiting dilutions consisting of 1.0 × 10^5^ and 5.0 × 10^5^ with five mice in each group (*n* = 10 BT853, *n* = 10 SU_MB002). Mice were assessed for both histological differences in tumor formation along with length of survival. Comparative histological and survival analyses of mice containing intracranial tumors generated from stable lines of SU_MB002 with β-catenin overexpression (*n* = 5) or negative-control construct expression (*n* = 5) were performed by using 1.0 × 10^4^ cells from each line. Tumor-initiating capacity, histological differences in tumor size, and survival analysis of SU_MB002 TGP+ (endogenous Wnt-active cells, *n* = 10) compared with SU_MB002 TGP− (endogenous Wnt-inactive cells, *n* = 10) were performed using five mice per dilution at dilutions of 1.0 × 10^4^, 5.0 × 10^4^. Tumor-initiating capacity and histological differences of HD-MB03 TGP+ compared with HD-MB03 TGP− cells were performed using two dilutions of 5 × 10^2^ and 1 × 10^3^ cells with 4 and 12 mice, respectively. L807mts treatment and PBS controls were delivered via intranasal injection to mice following brief anesthetization with isofluorane gas (2.5% induction). Treatment was given at 50 μg/dose every other day, following a 2-week engraftment incubation. Treatment and control mice were injected with 1.0 × 10^4^ cells for SU_MB002 (*n* = 12), 1.0 × 10^4^ cells for BT992 (*n* = 12), and 1.0 × 10^5^ cells for RCMB40 (*n* = 12). All mice were sacrificed at endpoint, brains were harvested, formalin-fixed, and paraffin-embedded for hematoxylin and eosin staining. Images were taken using the Aperio Slide Scanner and analyzed using ImageScope v11.1.2.760 software (Aperio).

For checking the radiosensitivity of L807mts in vivo, mice were intracranially xenografted with SU_MB002 cells at 60,000 cells per mouse (*n* = 16) and divided into four cohorts with *n* = 4/cohort: (i) Control, (ii) Radiotherapy; (iii) L807 and (iv) L807 + Radiotherapy. Ten days post injectection, mice allocated into treatment cohorts (iii) and (iv) were administered L807 intranasally at 50 μg/dose every other day for a total of six doses. Mice in groups (ii) and (iv) were given 2 Gy of craniospinal irradiation 14 days post injection. Following treatment completion, mice were monitored as per established animal utilization protocols and culled at endpoint.

### Immunoflourescence on xenografts

Five micrometers patient-derived xenograft paraffin-embedded tissues were deparaffinized in xylene and processed through a graded series of alcohol concentrations. Antigen retrieval was performed at 95–100 °C for 10 min in citrate buffer pH 6.0. Samples were then incubated with blocking solution (1% BSA, 0.2% Triton 100X, and 5% goat animal serum in 1x TRIS-buffered saline) for 45 min at the room temperature followed by overnight incubation in primary antibody, rabbit anti-human polyclonal Sox2 antibody (1:100, Abcam #97959) at 4 °C. The next day samples were washed in 1XTBS and treated with secondary antibody, Alexa Flour 488 goat anti-rabbit IgG (1:200, Life Technologies), for 2 h at the room temperature. Slides were then washed extensively with 1XTBS and counterstained with prolong gold anti-fade mountant with DAPI (Life technologies). Images were acquired using Olympus microscope and Volocity software. Nuclei from 20 high power fields were counted and positivity for Sox2 marker expression was quantified and expressed as % positive cells.

### Immunhistochemistry (IHC) on xenografts

Immunohistochemical staining was performed on 5 μM sections of TGP-negative and TGP-positive harvested tumor sections. Briefly, tumor sections were deparaffinized by immersion in xylenes followed by rehydration in increasing concentrations of alcohol (100, 95, 70%). Antigen retrieval was accomplished using a low pH solution (Vector laboratories) and a microwave. Prior to primary antibody incubation, endogenous peroxidases, biotin, biotin receptors, avidin binding sites, and nonspecific binding was blocked by treating slides with 3% hydrogen peroxide solution, Avidin/Biotin Blocking Kits (Vector Laboratories) and Normal Donkey Serum respectively. Rabbit polyclonal GFP primary antibody (Abcam; ab6556; 1:1000) incubation was performed overnight at 4 °C in a humidified chamber followed by donkey anti-rabbit biotin secondary antibody (Jackson ImmunoResearch Laboratories; 711-065-152; 1:1000) incubation at the room temperature for 2 h. Slides were washed in 1X TBS solution with or without 0.05% Tween-20 after each step. Following secondary antibody incubation, slides were stained with 3,3′-diaminobenzidine (Vector Laboratories), counterstained with hematoxylin, and differentiated with acid alcohol after which, slides were dehydrated in decreasing concentrations of alcohol (70, 95, 100%), cleared by immersion in xylenes and mounted with PolyMount (Polysciences Inc.). All images were obtained using the Aperio Slide Scanner (Leica Biosystems).

### Wnt3A-conditioned medium

L Wnt3A cells (ATCC #CRL-2647) transgenic mouse fibroblasts transfected with a Wnt3A expression vector, and control L cells (ATCC #CRL-2648) were cultured according to ATCC’s recommendations. Briefly, L Wnt3A cells were maintained in DMEM and 10% FBS with 0.4 mg/mL G418. Control L cells were maintained in DMEM and 10% FBS. Both, L Wnt3A and control L cells were subcultured 1:10 in culture medium without G418 and grown until confluency. Culture media were then removed and filtered (0.2 μm). Wnt3A and control conditioned media were separately mixed with BTIC enrichment medium at 1:1 for all subsequent experiments on primary human MB cells.

### Quantitative real-time polymerase chain reaction

Total RNA was extracted using the Norgen Total RNA isolation kit and quantified using a NanoDrop Spectrophotometer ND-1000. Complementary DNA was synthesized from 0.5 to 1 μg RNA using the qScript cDNA Super Mix (Quanta Biosciences) and a C1000 Thermo Cycler (Bio-Rad) with the following cycle parameters: 4 min at 25 °C, 30 min at 42 °C, 5 min at 85 °C, hold at 4 °C. qRT-PCR was performed using the Perfecta SybrGreen (Quanta Biosciences) and CFX96 instrument (Bio-Rad). CFX Manager 3.0 software was used for quantification of gene expression and levels were normalized to GAPDH expression with secondary validation normalized to Actin expression. Primers include: *Actin* (F: 5′-TATCCCTGTACGCCTCT-3′; R: 5′-AGGTCTTTGCGGATGT-3′), *Axin2* (F: 5′-TGGAGCCGGCTGCGCTTTGAT-3′; R: 5′-CTGGGGTCCGGGAGGCAAGTC-3′), *Bmi1* (F: 5′-GGAGGAGGTGAATGATAAAAGAT-3′; R: 5′-AGGTTCCTCCTCATACATGACA-3′), *GAPDH* (F: 5′-TGCACCACCAACTGCTTAGC-3′; R: 5′-GGCATGGACTGTGGTCATGAG-3′), *Sox2* (F: 5′-TCAGGAGTTGTCAAGGCAGAGAAG-3′; R: 5′-GCCGCCGCCGATGATTGTTATTAT-3′).

### Western immunoblotting

Ten micrograms of denatured total protein or 7.5 μg of nuclear and cytoplasmic extracts (NE PER reagents, Thermo Scientifc) was separated using 10% bis-tris gel electrophoresis and transferred to polyvinylidene fluoride membrane. Western blots were probed with the following primary antibodies: Bmi1 (mouse; 1:1000; Millipore #05-637), Sox2 (mouse; 1:2,000; BD Biosciences #561469), β-catenin (rabbit; 1:500; Cell Signaling #9581), HDAC1 (rabbit; 1:200 Millipore #06-720), β-tubulin (rabbit; 1:50,000; Abcam #ab6046), and GAPDH (mouse; 1:500; Abcam #ab8245). The secondary antibody was horseradish peroxidase conjugated goat anti-mouse IgG (Bio-Rad) or goat anti-rabbit IgG (Sigma). The bands were visualized using an Immobilon Western kit (Millipore) and Chemidoc (Bio-Rad).

### Lentiviral studies

β-catenin (#24313) overexpression and control (#12252) vectors and the TGP endogenous canonical Wnt reporter (8X TOPFlash TCF reporter) (#24305) were purchased from Addgene. Replication-incompetent lentviruses were produced by cotransfection of the expression vector and packaging vectors pMD2G and psPAX2 in HEK293FT cells. Viral supernatants were harvested 72 h after transfection, filtered through a 0.45 μm cellulose acetate filter, and precipitated with PEGit (System Bioscences). The viral pellet was resuspended in 1.0 mL of DMEM media and stored at −80 °C. Stable cell lines were generated by transduction followed by maintenance of cultures with puromycin or flow cytometric cell sorting for GFP+ and GFP− cells.

### Flow cytometric analysis and cell sorting

Tumorspheres were dissociated and single cells resuspended in PBS. Samples were sorted using a MoFlo XDP Cell Sorter (Beckman Coulter). Dead cells were excluded using the viability dye 7AAD (1:10, Beckman Coulter) or using a near IR Live/Dead fixable staining kit (Life Technologies). Compensation was performed using mouse IgG CompBeads (BD). GFP expression was defined as positive or negative based on the analysis of regions established by the isotype control. Cells were sorted into tubes containing 1 mL BTIC enrichment medium and small aliquots from each sort tube were analyzed to determine the purity of the sorted populations. Cells were allowed to equilibrate at 37 °C for a few hours prior to experimentation. Intracellular levels of Bmi1 and Sox2 were determined following preparation using Fixation/Permeabilization Solution Kit (Cat # 554714, BD Biosciences) along with antibodies anti-Bmi1 (1:11; Cat # 130-106-736, Miltenyi) and anti-Sox2 (1:20; Cat # 561610, BD Horizons), for which viable cells were stained using LIVE/DEAD™ Fixable Near-IR Dead Cell Stain Kit (Cat # L10119, Thermo Scientific).

### Small molecule Wnt activation

The competitive ATP inhibitor that functions as a selective GSK-3 inhibitor, CHIR99021, was used to activate the canonical Wnt pathway in SU_MB002 and ICB1299 cells. Similarly, the substrate-competitive peptide inhibitor of GSK-3, L807mts, was also used to activate the canonical Wnt pathway in SU_MB002 and ICB1299 cells. 2 × 10^5^ cells were plated in a 24-well plate in triplicate at a volume of 500 μL/well at CHIR99021 concentrations of 1, 3, 5, 7, 10 μM. DMSO was used as a control. CHIR99021 and DMSO were replenished after 24 and 48 h and cultures were used for qRT-PCR, self-renewal, proliferation, or radiation assays after 3 days. 2 × 10^5^ cells were plated in a 24-well plate in triplicate at a volume of 500 μL/well at L807mts concentrations of 1, 5, 10 μM. PBS was used as a control. L807mts and PBS were replenished after 24 and 48 h and cultures were used for qRT-PCR, self-renewal, and proliferation.

### In vitro radiotherapy

Cells were plated at density of 1 × 10^6^ cells/mL and treated with either one dose of 2 Gy radiation (Faxitron RX-650) and incubated for a week or a range of doses ranging from 0 to 5 Gy. Functional self-renewal and proliferation assays were performed following incubation.

### Single-cell RNA-seq and library preparation

Single-cell suspensions of 1000 cells with a final viability of >80% was used. Single-cell library preparation was carried out as per the 10X Genomics Chromium single-cell protocol using the v2 chemistry reagent kit (10X Genomics, Plesanton, CA, USA). Cell suspensions were loaded onto individual channels of a Chromium Single-Cell Chip along reverse transcription (RT) master mix and single-cell 3′ gel beads, aiming for 1000 captured cells per sample. Following generation of gel bead-in-emulsions (GEMs), cDNA was underwent a two-stage purification process with Dynal MyONE Silane beads (Thermo Fisher Scientific), followed by SPRISelect beads (Beckman Coulter). RT and cDNA amplification were performed using a 96-well Veriti Thermocycler (Life Technologies). Amplified cDNA purified using SPRIselect beads and sequencing libraries were generated with unique sample indexes. Dual sided SPRIselect cleanup was used to clean up samples and size select. Libraries were sequenced on an Illumina 2500 in Highoutput mode at the Princess Margaret Genomics Centre (Toronto, ON) using the 10X Genomics recommended sequencing parameters. Samples were quantified by Kapa Library Quantification kit (Roche) and normalized to achieve the desired median read depth per cell.

### Single-cell RNA-seq data preprocessing

The Cell Ranger software pipeline (version 2.1.0) developed by 10X Genomics was used to demultiplex cell barcodes, map reads to the transcriptome (GRCh38) using STAR aligner and down-sample reads as required to generate normalized aggregate data across samples. The number of reads per cell barcode was calculated using the BamTagHistogram function in the Drop-seq Alignment Cookbook^49^. Subsequently, the number of cells per sample was determined by calculating the cumulative fraction of reads corresponding to each individual cell barcode in a library. Cell barcodes were sorted in decreasing order and the inflection point was identified using the R package Dropbead^50^ (version 0.3.1) to distinguish between empty droplets with only ambient RNA and true droplets containing a cell. The raw matrix of gene counts versus cells from Cell Ranger output was filtered by cell barcodes identified from Dropbead. We processed the resultant unique molecular identifier (UMI) count matrix using the R package Seurat^51^ (version 2.3.4).

### Single-cell RNA-seq quality control, normalization, and dimensionality reduction

For each cell, we quantified three quality control metrics: the number of genes with at least one UMI, the total number of UMIs detected and the percentage UMI counts belonging to expressed mitochondrial genes. We excluded all cells with >30% mitochondrial UMIs, potentially indicative of damaged cells with compromised cellular membranes.

Similar to the workflow in Lun et al.^52^, likely multiplet captures were removed if log-library size exceeded 2 median absolute deviations above the median. We further discarded low-quality cells where below 500 genes were detected. We filtered out lowly expressed genes detected in less than 5 cells across the aggregated dataset, corresponding to 1% of cells in the smallest library. After applying these QC criteria, 3725 single cells and 18721 genes remained and were included in downstream analysis. Expression normalization was performed in Seurat on the filtered matrix to obtain log-normalized counts scaled to library size. Relative gene expression was calculated by centering expression across all cells in the cohort using the ScaleData() function in Seurat. Identification of highly variable genes (4140 genes), PCA (11 signicificant PCs determined by a scree plot) and SNN-Cliq-inspired clustering were performed in Seurat to generate t-distributed stochastic neighbor embedding (t-SNE) visualizations (Rtsne: Use the Rtsne package Barnes-Hut implementation of tSNE; default in Seurat).

### Single-cell RNA-seq gene signature scoring and classification

Gene signature scores in individual cells were calculated with two methods. The first being the AddModuleScore() function in the R package Seurat (version 2.3.4). In brief, the average relative expression level for each gene signature was calculated on a single-cell level and subtracted by the aggregated relative expression of control gene sets. Control gene sets were defined by binning all 18,721 expressed genes into 25 bins of aggregate expression levels and then for each gene in the gene signature of interest, randomly selecting 100 genes from the comparable expression bin. As a result, control gene sets have comparable expression level distributions to the gene signature of interest. A second set of gene signature enrichment scores were calculated using GSVA with default parameters.

To estimate the significance of gene signature scores, a hundred sets of randomly selected genes sets with the same size as the gene signatures were generated and scored using both methods described above. Random sets were then used to define a 5% cutoff for the expected gene signature scores. Cells were classified as “enriched” for a given signature if they surpassed the 5% threshold.

### Single-cell RNA-seq analysis of publicly available data

Normalized count matrices for human MB tumor scRNA-seq data for 25 samples (five Wnt, three Shh, eight Group 3, nine Group 4) accounting for 7745 malignant cells was downloaded from GSE119926^27^. Normalized log2(TPM/10 + 1) expression was transformed into log(TPM/10 + 1) for input into Seurat to perform dimensionality reduction and clustering as described previously (6571 variable genes, 15 significant PCs). Cells were scored for gene signatures with GSVA and classified as previously described.

scRNA-seq data in the form of raw UMI count matrices for eight human MBs (two Shh, two Group 3, four Group 4) accounting for 28,268 cells were downloaded from Vladoiu, et al.^28^. Raw counts were log normalized using Seurat (v2.3.4). Data scaling, dimensionality reduction, and clustering were performed as described previously (2814 variable genes, 12 significant PCs) using Seurat. Cells were scored for gene signatures with GSVA (v1.30.0) and classified as previously described. Microglia/monocyte (AIF1, CD14, CD68) and T cell (CD3G, CD8A, IL7R) clusters were annotated using known gene markers and cell labels as described in Vladoiu, et al.^28^. Remaining cells labeled as malignant tumor cells (1155 microglia/monocytes; 188T cells; 26,925 tumor cells).

### Gene set variance analysis of publicly available data

Microarray data for Cavalli et al.^10^ were obtained from GEO (GSE85217). ENSEMBL gene identifiers were converted to HGNC symbols with annotations downloaded from biomart. HGNC symbols mapping to an identical ENSG ID were handled by only taking 1 HGNC symbol per ENSG id, and ESNG IDs mapping to an identical HGNC symbol were handled by only using 1 ENSG per HGNC symbol. GSVA 3 signatures were used for GSVA scoring: Group 3 (*n* = 7941 genes) and Group 4 (*n* = 6510 genes) defined in differential expression analyses (Group 3 vs. union of Wnt and Group 4, Group 4 vs. union of Wnt and Group 3, respectively), and the Broad Institute MSIGDB HALLMARK_WNT_BETA_CATENIN_SIGNALING signature (*n* = 42 genes). Differential expression analyses were performed using limma version 3.38.3. GSVA was run using GSVA version 1.30.0.

Microarray data from Northcott et al.^1^ were generously provided by the Northcott laboratory. ENSEMBL transcript ids (ENST) were mapped to HGNC symbols in a manner similar to that done for mapping ENSG ids to HGNC symbols for the Cavalli et al.^10^ data. GSVA scoring was done using the same methods as for the Cavalli et al.^10^ data.

### Statistical analysis

At least three biological replicates were performed for each experiment. Data represent mean ± standard error (mean) with *n* values listed in figure legends. Student *t* test analyses were performed using GraphPad Prism™ with significance set to *P* < 0.05. Kaplan–Meier survival curves were visualized using GraphPad Prism™. All statistical analysis and data visualization for scRNA-seq was performed in R (version 3.5.0).

### Reporting summary

Further information on research design is available in the Nature Research Reporting Summary linked to this article.

## Supplementary information

Supplementary Information

Description of Additional Supplementary Information

Supplementary Data 1

Supplementary Data 2

Reporting Summary

## Data Availability

The RNA-Seq data discussed in this publication have been deposited in NCBI’s Gene Expression Omnibus and are accessible through GEO Series accession number GSE131473. The scRNA-seq data have been deposited in CReSCENT (https://crescent.cloud/ CRES-P22) and Processed data is uploaded on BROAD institute site https://singlecell.broadinstitute.org/single_cell/study/SCP840. Source data are provided with this paper.

## Source data

Source Data
