# Supplementary material for: Wnt activation as a therapeutic strategy in medulloblastoma
Source: Nat Commun. 2020 Aug 28;11:4323. doi: 10.1038/s41467-020-17953-4 (PMC7455709; doi:10.1038/s41467-020-17953-4)
Supplement: Supplementary file 1 — Supplementary Information [file 41467_2020_17953_MOESM1_ESM.pdf]

# Wnt activation as a therapeutic strategy in medulloblastoma

**Manoranjan et al.**

**Supplementary Information**

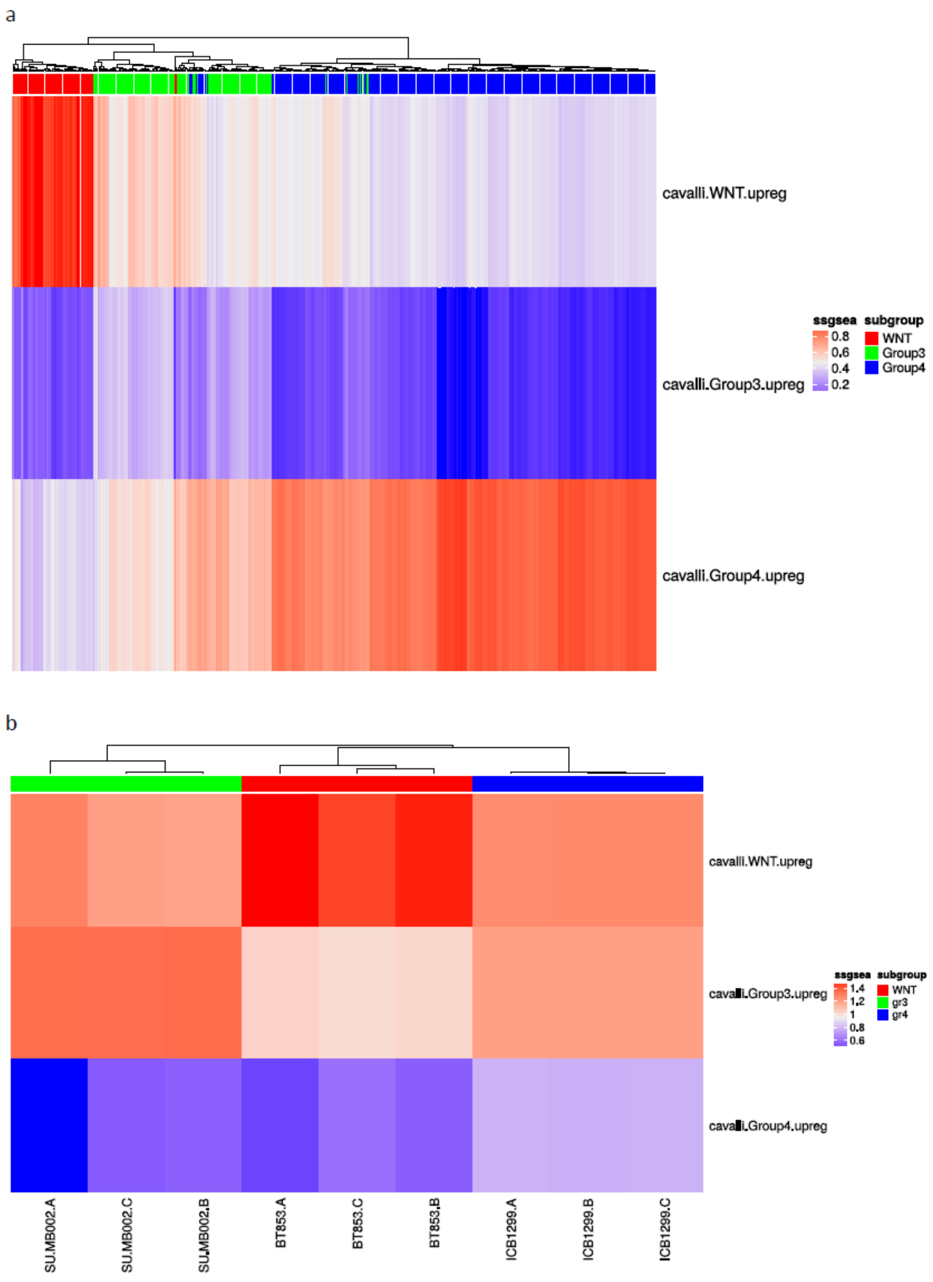


**Supplementary Figure 1: MB BTIC lines maintain their subgroup affiliation.** (a) Heatmap of 540 MB samples (GSE85217) scored for relative expression of MB subgroup upregulated gene expression signatures, computed from the same data. MB subgroup gene signatures (rows) are genes that are upregulated for a given MB subgroup relative to all other samples in this dataset. Signatures included all upregulated genes passing an

FDR threshold of 0.05 in this comparison. Samples were scored for expression of these signatures using ssGSEA. ssGSEA score is plotted as color, with better matches being more red and worse matches more blue, as per legend. Sample group classification is plotted as a color bar along the top of the heatmap, as per legend. (b) Heatmap of 9 MB stem cell lines from our current study, scored for relative expression of WNT, Group 3, and Group 4 MB subgroup upregulated gene signatures defined in panel A, using ssGSEA. Interestingly, the WNT, Group 3, and Group 4 MB signatures showed strongest relative enrichment in our WNT (BT853), Group 3 (SU_MB002), and Group 4 (ICB1299) BTIC lines, respectively, showing that the subgroup level differences seen in bulk tumors are preserved in our model system. Heatmap visualization is as in (a).

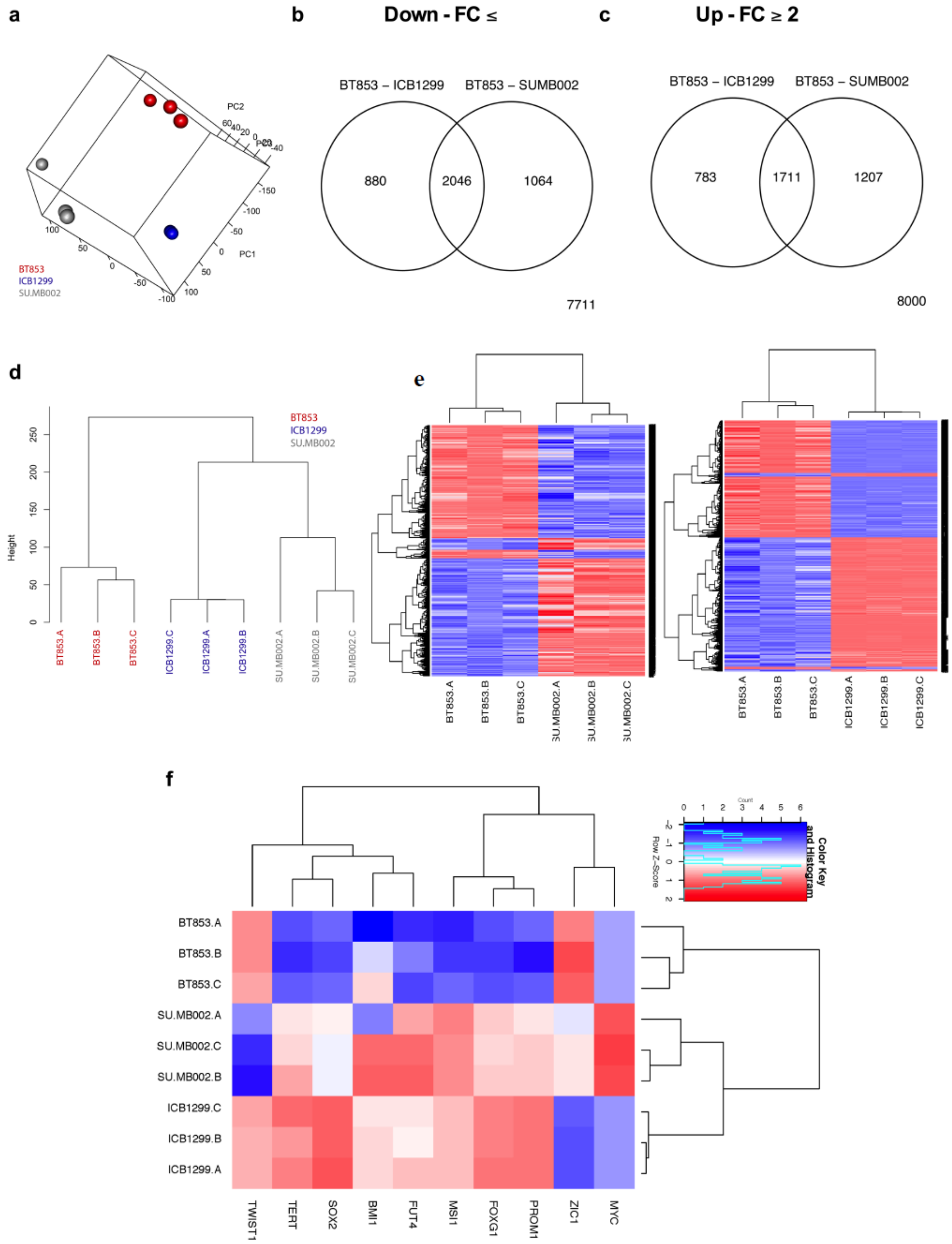


**Supplementary Figure 2: Wnt MB patient-derived lines maintain distinct transcriptional profile from Group 3 and Group 4 MB lines.** (a) Visualization of the first 3 principal components from PCA of Wnt (BT 853), Group 3 (SU_MB002), and Group

4 (ICB1299) primary patient-derived MB lines (n=3, independent samples per MB line; 1 line per subgroup). Venn diagrams of differentially (b) downregulated and (c) upregulated genes between Wnt/Group 3 and Wnt/Group 4 comparisons (n=3, independent samples per MB line, 1 line per subgroup, FC = fold change). (d) Dendrogram of Wnt, Group 3, and Group 4 samples submitted for RNA-seq (n=3, independent samples per MB line, 1 line per subgroup). (e) Heatmaps of differentially expressed genes between Wnt and Group 3 (left panel) or Wnt and Group 4 (right panel) samples (n=3, independent samples per MB, 1 line per subgroup). (f) Heatmap of key genes reported to be enriched in malignant and non-cancerous stem cell populations are significantly upregulated in Group 3 and 4 MB lines when compared to Wnt MB (n=3, independent samples per MB line; 1 line per subgroup).

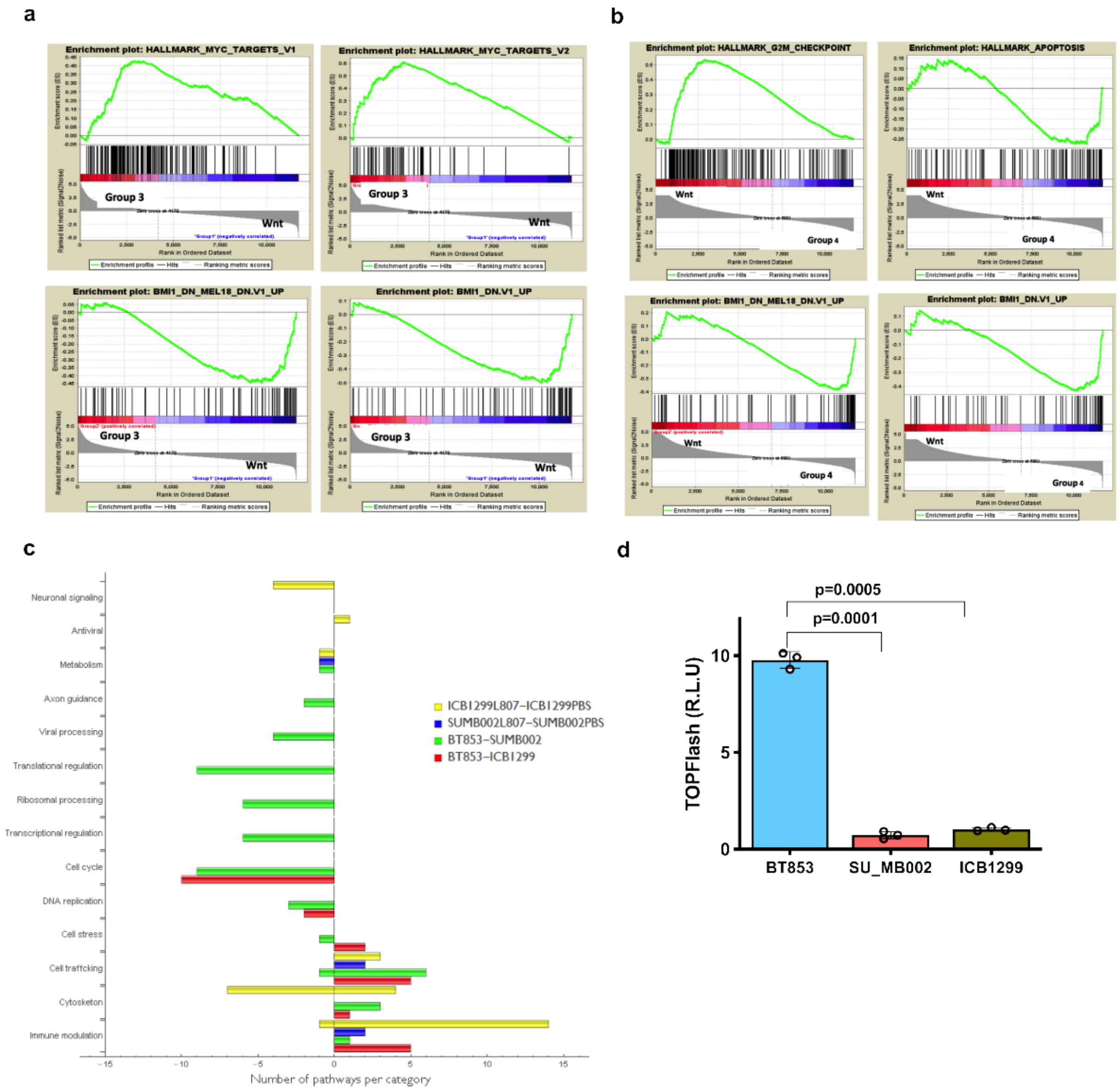


**Supplementary Figure 3: Group 3 and 4 MB lines display enriched expression in oncogenic pathways compared to Wnt MB.** (a) Myc targets and Bmi1 associated genes were significantly enriched in Group 3 when compared to Wnt MB by GSEA. (b) Similarly, Bmi1 associated genes were enriched in Group 4 when compared to Wnt MB. Further, cell cycle inhibitors and apoptotic factors were enriched in Wnt when compared to Group 4 MB. (c) Pathways found to be significantly regulated between Wnt, Group 3, Group 4, L807mts-treated and control samples (n=3, independent samples per MB line,

1 line per subgroup). Pathways were grouped based on biological categories. Each bar indicates a number of pathways found to be significantly regulated in a given comparison belonging to the category of interest. (d) Differential Wnt reporter activity between Wnt and Group 3 (p=0.0001) or Group 4 MB (p=0.0005) (n=3, independent experiments per MB line; 1 line per subgroup). Error bars expressed as mean ± standard error (mean) using two-tailed, unpaired Student's t-test).

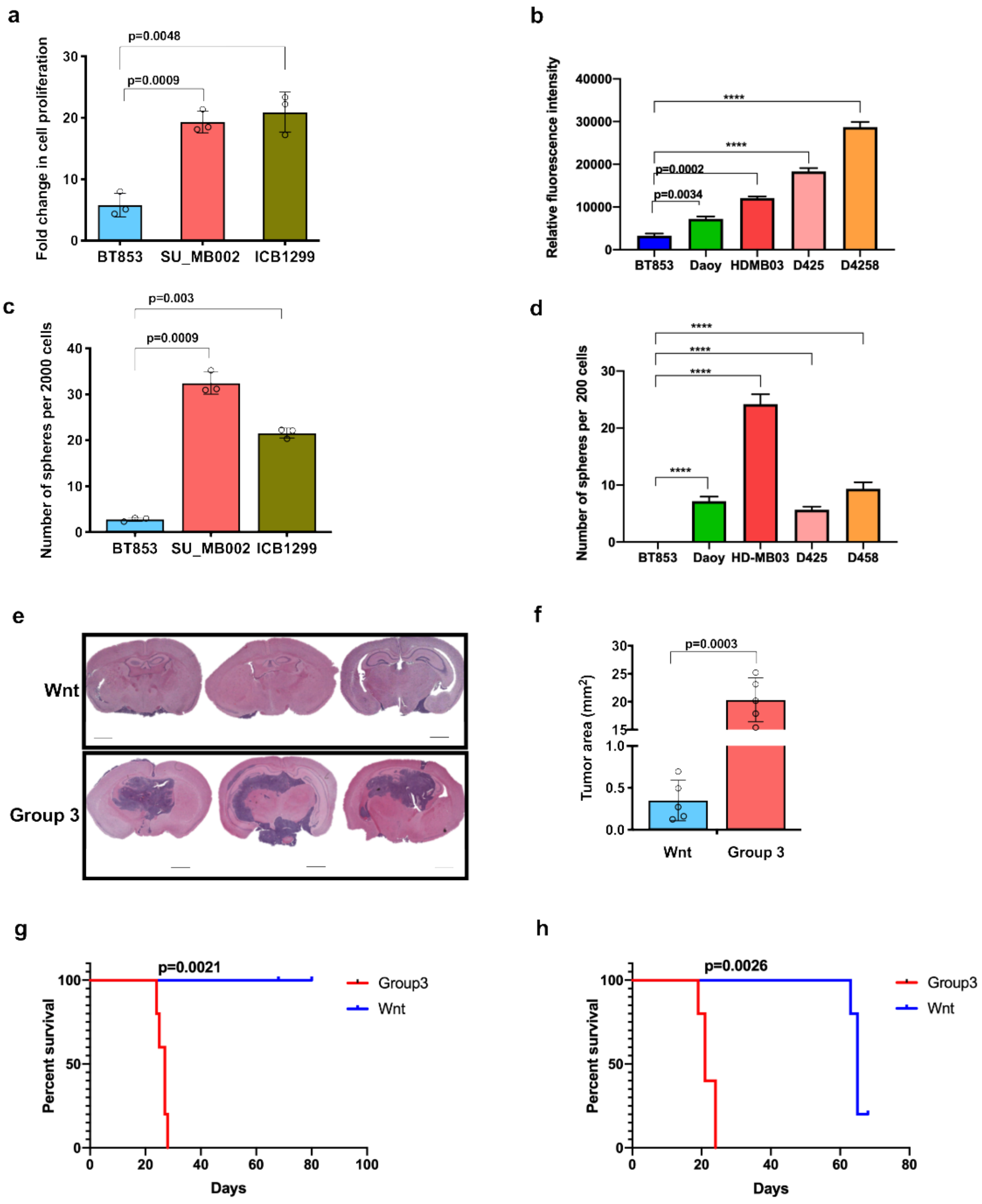


**Supplementary Figure 4: Wnt-activated MB is less tumorigenic than Shh, Group 3, and Group 4 MB lines.** (a) Wnt MB (BT853) displays a reduced proliferative capacity when compared to Group 3 (SU_MB002 (p=0.0001)) and Group 4 MB (ICB1299 (p=0.0005)) (n=3, independent experiments per MB line). (b) Wnt MB (BT853) displays a reduced proliferative capacity when compared to Shh MB (Daoy (p=0.0034)) and Group

3 MB (HD-MB03 (p=0.0002), D425 (p=0.000007) and D458 (p=0.000002); n=4 independent experiments per cell line). (c) Wnt MB (BT853) maintains a reduced self-renewal capacity when compared to Group 3 (SU_MB002 (p=0.0009)) and Group 4 MB (ICB1299 (p=0.0048)) (n=3, independent experiments per MB line). (d) Wnt MB (BT853) maintain a reduced self-renewal capacity when compared to Shh MB (Daoy (p=0.000004)) and Group 3 MB (HD-MB03 (p=0.00000009), D425 (p=0.000001) and D458 (p=0.00001); n=6 independent experiments per MB line). (e) Representative histology images of Wnt (BT853) and Group 3 (SU_MB002) MB xenografts demonstrate significant tumor reduction in Wnt (n=10 mice) compared to Group 3 MB xenografts (n=10 mice). (f) Tumor volume in Wnt (BT853) xenografts (n=10 mice) is significantly reduced compared to Group 3 (SU_MB002) xenografts (n=10 mice) (p=0.0003). Wnt MB (BT853) xenografts (n=5 mice for each dilution) display a significant increase in overall survival when compared to Group 3 (SU_MB002) xenografts (n=5 mice for each dilution) at cell dilutions of (g) $1.0x10^5$ (p=0.0021; median survival Group 3: 27 days, Wnt: undefined) and (h) and $5.0x10^5$ (p=0.0026; median survival Group 3: 21 days, Wnt: 65 days). Histology image scale bar=5000 µm. Figures (a), (b), (c), (d), and (f) contain error bars expressed as mean ± standard error (mean) using two-tailed, unpaired Student's t-test. Figures (g) and (h) analyzed using log-rank (Mantel-Cox) test. **** p<0.0001.

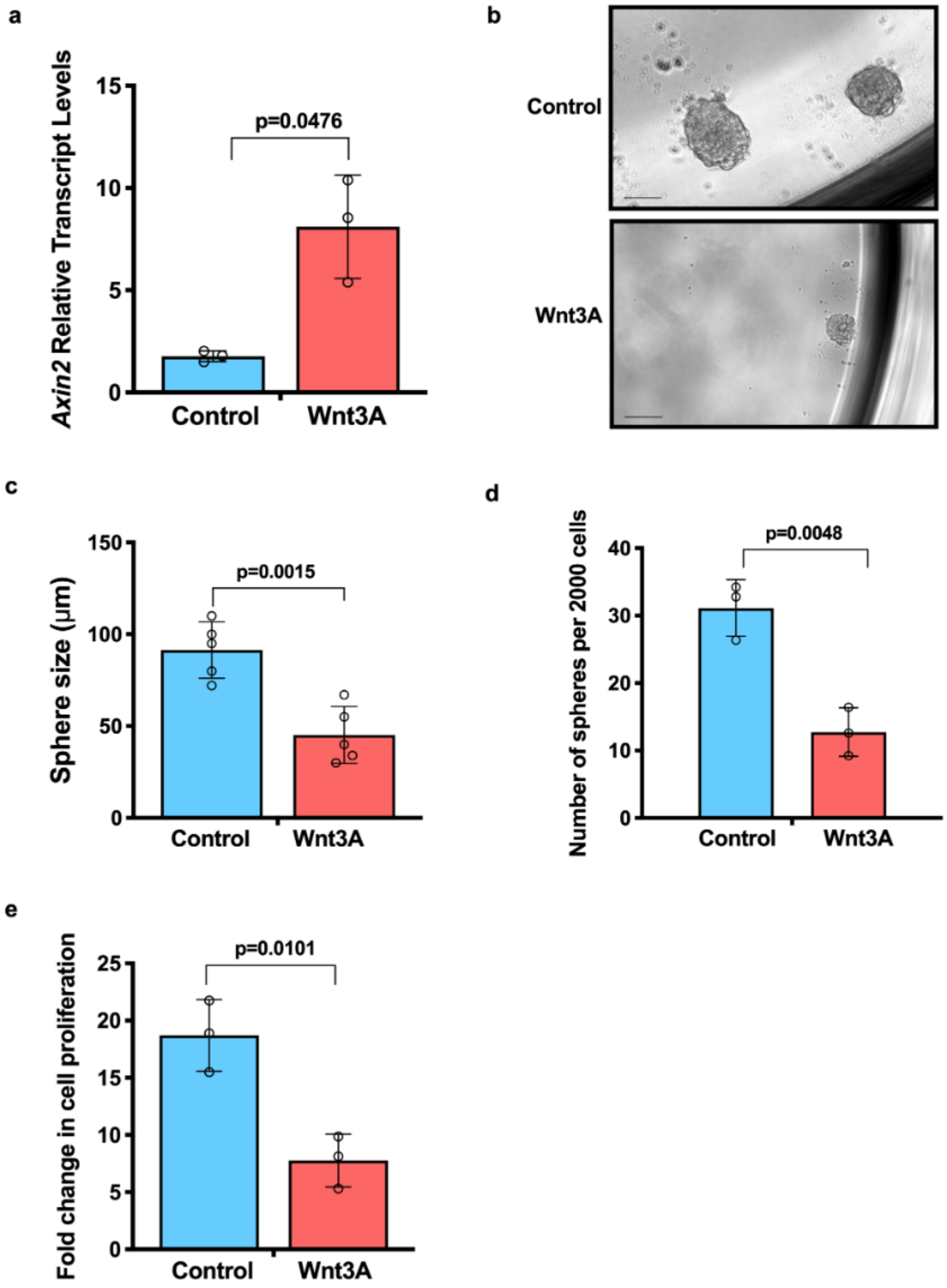


**Supplementary Figure 5: Wnt3A-conditioned medium reduces *in vitro* stemness in treatment-refractory Group 3 MB line.** (a) *Axin2* transcript levels are enriched in SU_MB002 cells treated with Wnt3A-conditioned medium compared to control (p=0.0476) (n=3, independent experiments). (b) Light microscopic images of tumor spheres treated with control and Wnt3A-conditioned medium (scale bar = 200 µm). (c)

Wnt3A-conditioned medium-treated cells display a significant reduction in tumor sphere size ($p=0.0015$) ($n=3$, independent experiments). (d) Self-renewal ($p=0.0048$) and (e) proliferative ($p=0.0101$) capacity of MB stem cells are significantly reduced following culture with Wnt3A-conditioned medium when compared to control ($n=3$, independent experiments). Figures (a), (c), (d), and (e) contain error bars expressed as mean ± standard error (mean) using two-tailed, unpaired Student's t-test.

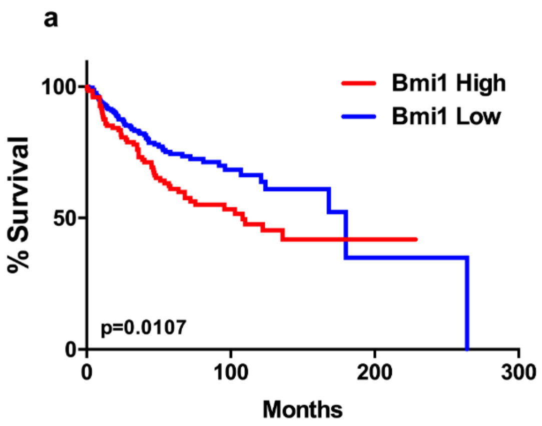


**Supplementary Figure 6: Enriched expression of Bmi1 and Sox2 gene signatures is associated with poor outcome in Group 3 and 4 MB patients.** Kaplan-Meier survival plots show survival of combined Groups 3 and 4 patients (n=377) based on (a) the *Bmi1* signature (median survival: *Bmi1*$^{high}$ 103.3 months, *Bmi1*$^{low}$ 180.0 months; p=0.0107) and (b) *Sox2* signature (median survival: *Sox2*$^{high}$ 110 months, *Sox2*$^{low}$ 168.0 months; p=0.004). Data analyzed using log-rank (Mantel-Cox) test.

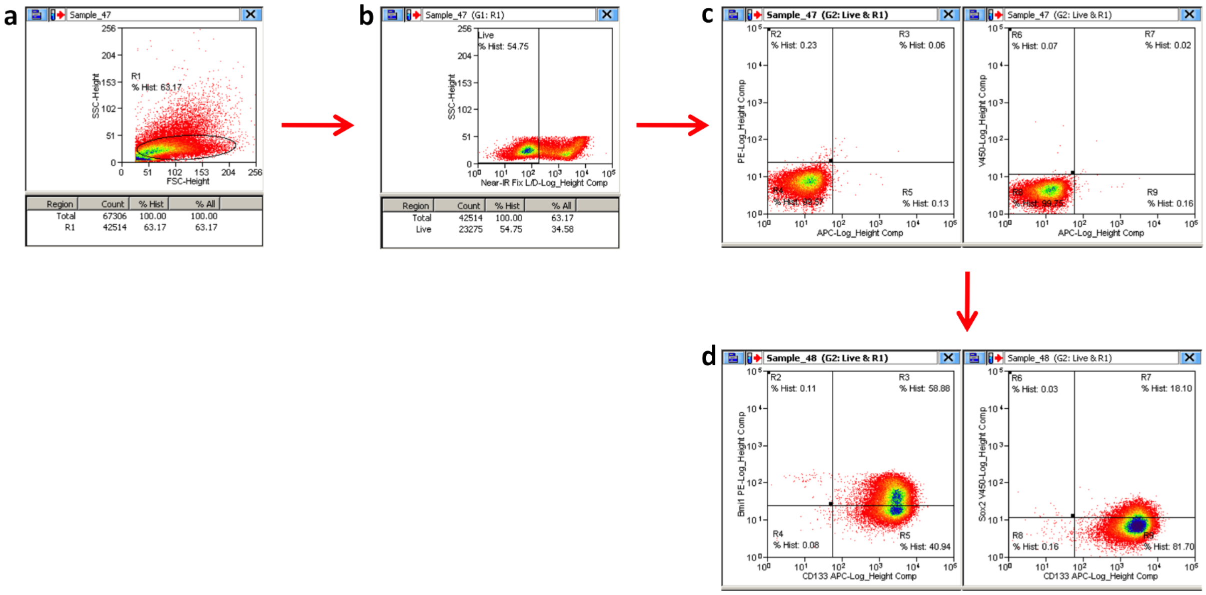


e

| Subgroups | Samples | BMI1 | Sox2 |
|---|---|---|---|
| WNT | BT853 | 0.40% | 0.11% |
| SHH | Daoy | 66.90% | 85.49% |
| Group 3 | HD-MB03 | 99.90% | 97.74% |
| | RCMB-40 | 100.00% | 0.45% |
| | D425 | 57.10% | 0.54% |
| | Med8A | 44.32% | 89.47% |
| | D458 | 81.07% | 1.61% |
| Group 4 | ICB1299 | 58.99% | 18.13% |

**Supplementary Figure 7: Group 3 and Group 4 MBs express higher levels of Sox2 and Bmi1 compared to Wnt MB.** (a) Flow gating strategy: FSC-Height vs. SSC-Height is used as the initial gate to exclude debris. (b) Viability gate is set using Live/Dead Fixable Near-IR stain to exclude non-viable cells. (c) Unstained control is used to set the gate for expression of Bmi1, CD133 and Sox2, where gate is drawn to exclude baseline expression of fluorophores. (d) Sample stained with antibodies show expression of Bmi1, CD133, and Sox2. (e) Flow analysis shows higher expression of Sox2 and Bmi1 in Shh (Daoy) and Group 3 (HD-MB03, RCMB40, D425, Med8A, D458), and Group 4 (ICB1299) MB lines when compared to Wnt (BT853).

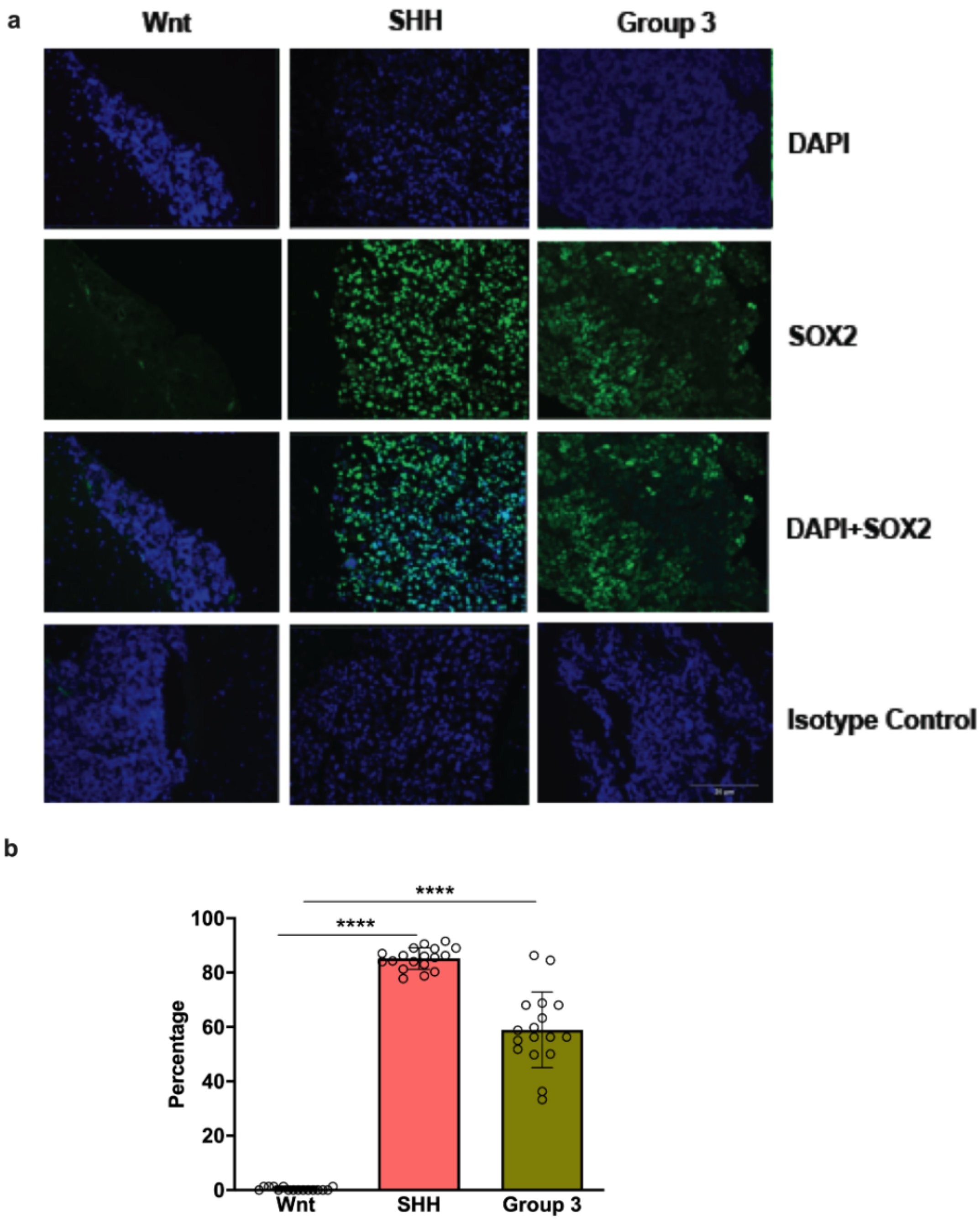


**Supplementary Figure 8: Shh and Group 3 MBs express higher levels of Sox2 when compared to Wnt MB.** (a) Representative image of xenografts from Wnt (BT853), Shh (Daoy), and Group 3 (SU_MB002) MB lines stained for Sox2. (b) Quantification of IF staining showing Shh (Daoy, $p<1.0\times10^{-10}$) and Group 3 (SU_MB002, $p<1.0\times10^{-10}$) MB

lines have higher Sox2 expression compared to Wnt (BT853) MB (n=16, independent samples per MB line, 1 line per subgroup). Error bars expressed as mean ± standard error (mean) using two-tailed, unpaired Student's t-test. Scale bar = 20 μm. **** $p<0.0001$.

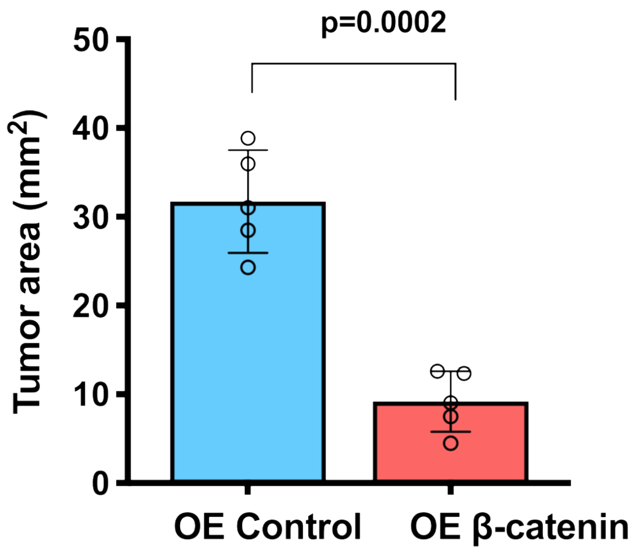


**Supplementary Figure 9: Wnt activation through ectopic mechanisms reduce overall tumor burden.** (a) Xenografts generated with cells ectopically expressing of β-catenin (n=5 mice) contain smaller tumor volumes when compared to control xenografts (n=5 mice) (p=0.0002). Error bars expressed as mean ± standard error (mean) using two-tailed, unpaired Student's t-test.

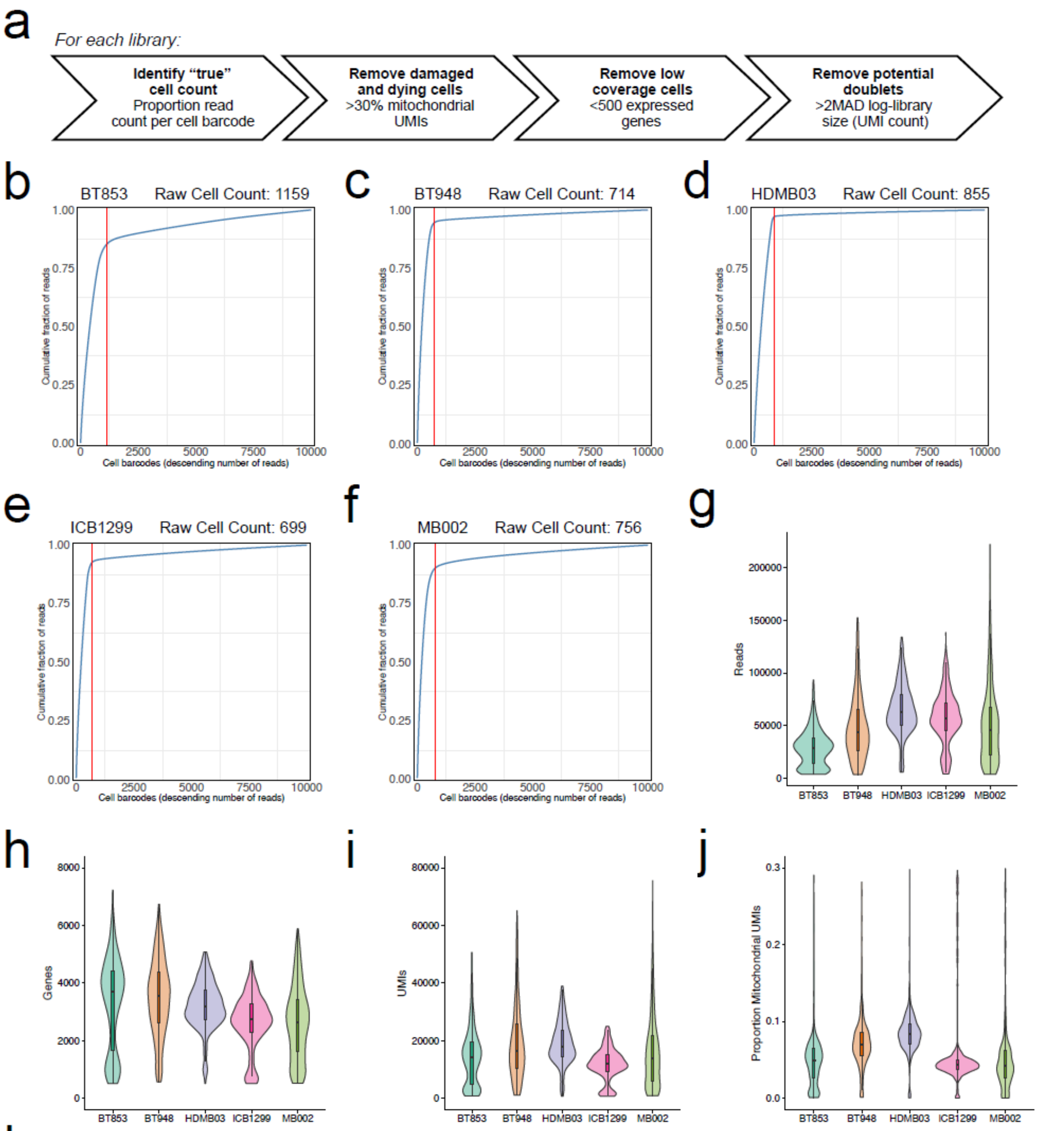


k

| Sample ID | Subtype | Raw Cell Count | Cell Count Post-QC | Median Reads | Median UMIs | Median Genes | Average Percent Mito |
|---|---|---|---|---|---|---|---|
| BT853 | Wnt | 1159 | 1099 | 28520 | 14188 | 3693 | 4.9 |
| BT948 | Group 4 | 714 | 642 | 43559 | 16482 | 3553 | 7.1 |
| HDMB03 | Group 3 | 855 | 813 | 62911 | 18024 | 3178 | 8.5 |
| ICB1299 | Group 4 | 699 | 553 | 56827 | 11856 | 2750 | 5.6 |
| MB002 | Group 3 | 756 | 619 | 45568 | 13723 | 2630 | 5.2 |
| **Total:** | 3,725 high-quality cells from 5 patient-derived MB BTIC cultures. 18,721 genes detected across the cohort. | | | | | | |

**Supplementary Figure 10: Quality control of scRNA-seq data to eliminate potential doublets, damaged and low quality cells.** (a) Analysis pipeline to identify suspect cells. (b-f) Determining cell number from scRNA-seq libraries using droplet read counts. Cell

barcodes ordered by increasing read number. Inflection point represents cell number in library. Cell barcodes to the right of the inflection point contribute minimally to total read counts and are likely empty droplets with ambient RNA. Final QC metrics of scRNA-seq data post-filtering displaying (g) number of reads per cell, (h) number of genes per cell, (i) number of transcripts / UMIs per cell and (j) proportion mitochondrial reads per cell. (k) Sequencing metrics summarized as a table for the final, high quality cohort of 3725 cells and 18721 genes across 5 samples.

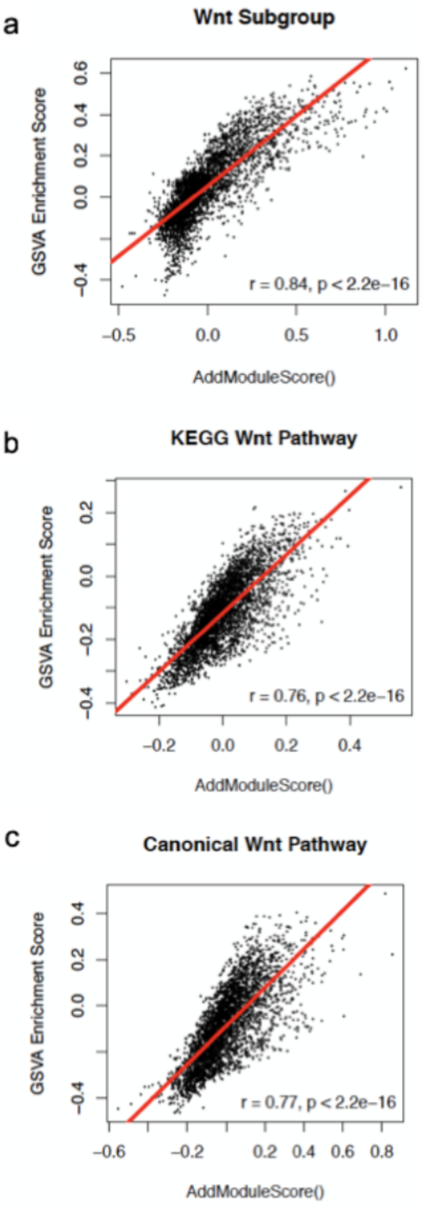


**Supplementary Figure 11: Comparison of gene signature scoring methods for scRNA-seq data.** (a-c) Relative expression of individual cells was scored for 8 gene signatures using two methods (1) AddModuleScore() within Seurat and (2) GSVA. Scores from the two methods were compared at a single cell level, each point is a cell (n=3725 cells from 5 samples). The two methods have a high degree of correlation across the 3 signatures, assessed by spearman correlation coefficient (r). These findings suggest that

the scoring method presented in the previous submission is valid and does not overinflate gene signature values of individual cells. Given that the p-value is extremely small and not returned by the Spearman correlation function, an exact value cannot be provided, $p<2.2x10^{-16}$.

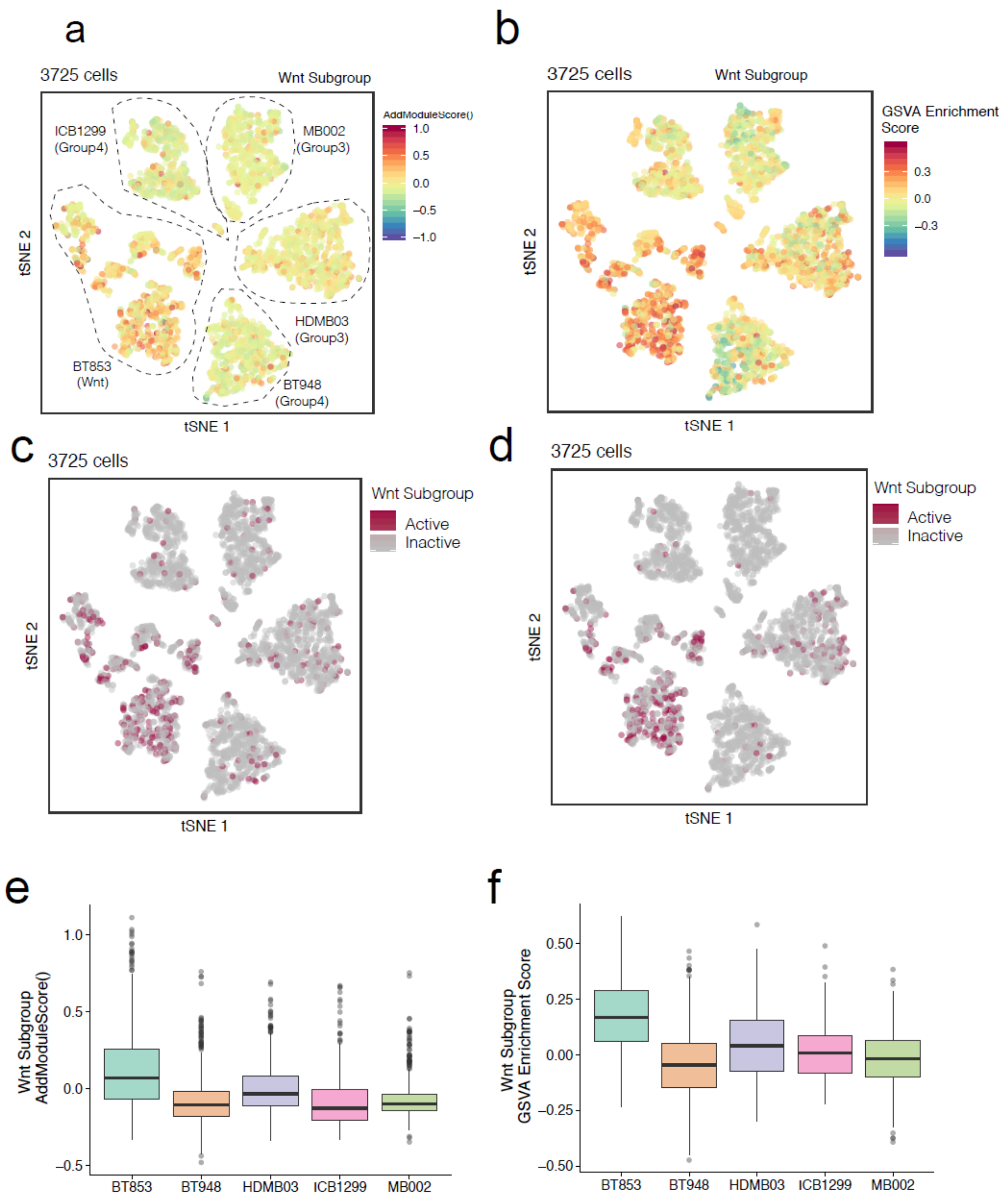


**Supplementary Figure 12: Visually comparing gene signature scoring methods for scRNA-seq data.** Regardless of the scoring method used, the Wnt Subgroup sample has the highest enrichment for Wnt Subgroup active cells and subsequent gene signature values. (a-c) AddModuleScore (d-f) GSVA enrichment score. Box plots represent the

median, upper, and lower quartiles of the distribution and whiskers represent 1.5x IQR or the most extreme value. Outliers represented as circles. n=3725 cells across 5 samples.

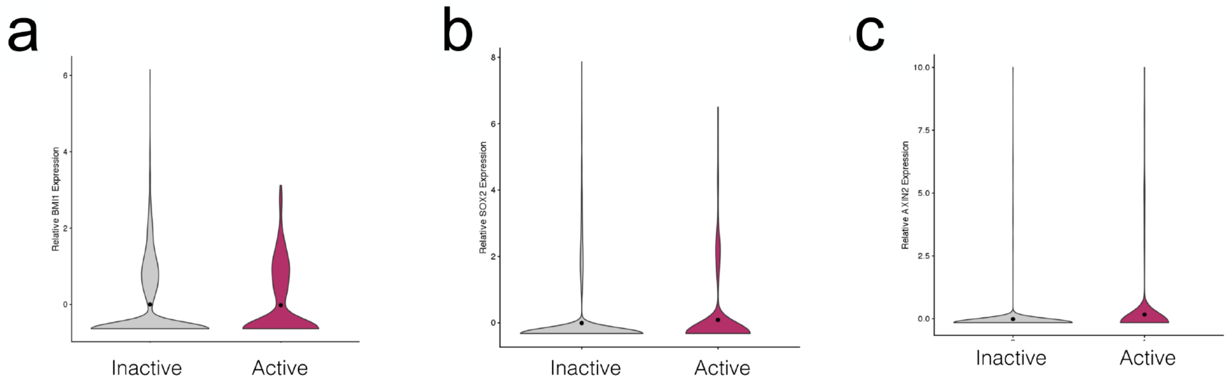


**Supplementary Figure 13:** Relative expression of (a) *BMI1* (b) *SOX2* and (c) *AXIN2* in Wnt active cells compared to Wnt inactive cells as deduced by scRNA seq.

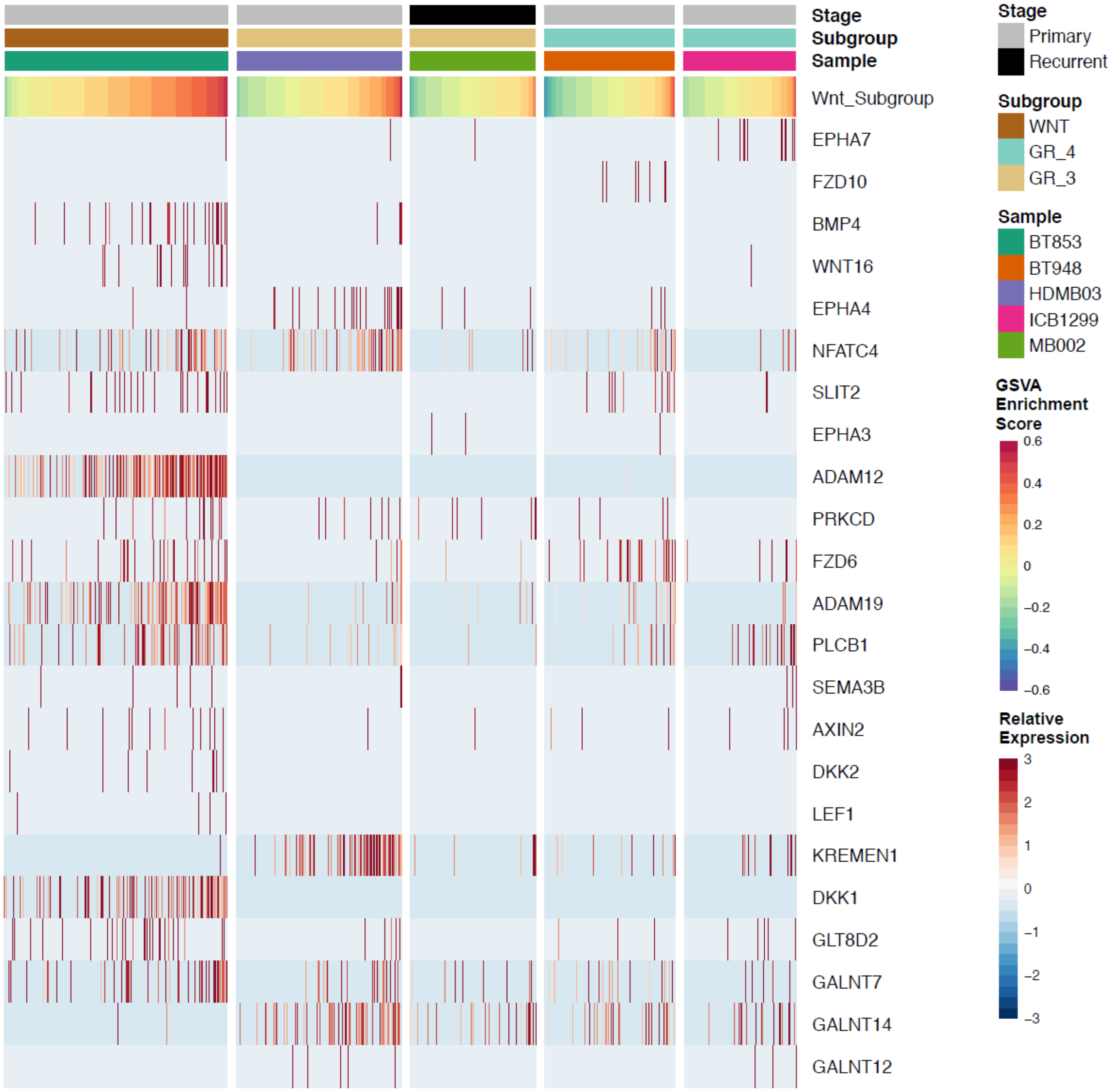


**Supplementary Figure 14: Wnt signature is present in a minority of Group 3 and 4 MB cells.** Heatmap of Wnt MB scoring for RNA seq performed on individual cells obtained from 5 patient-derived MB lines, including Wnt, Group 3, and Group 4 MBs. Gene expression (rows) represented by relative expression (Z scores). Cells (columns) are sorted by increasing Wnt subgroup GSVA enrichment score within each sample.

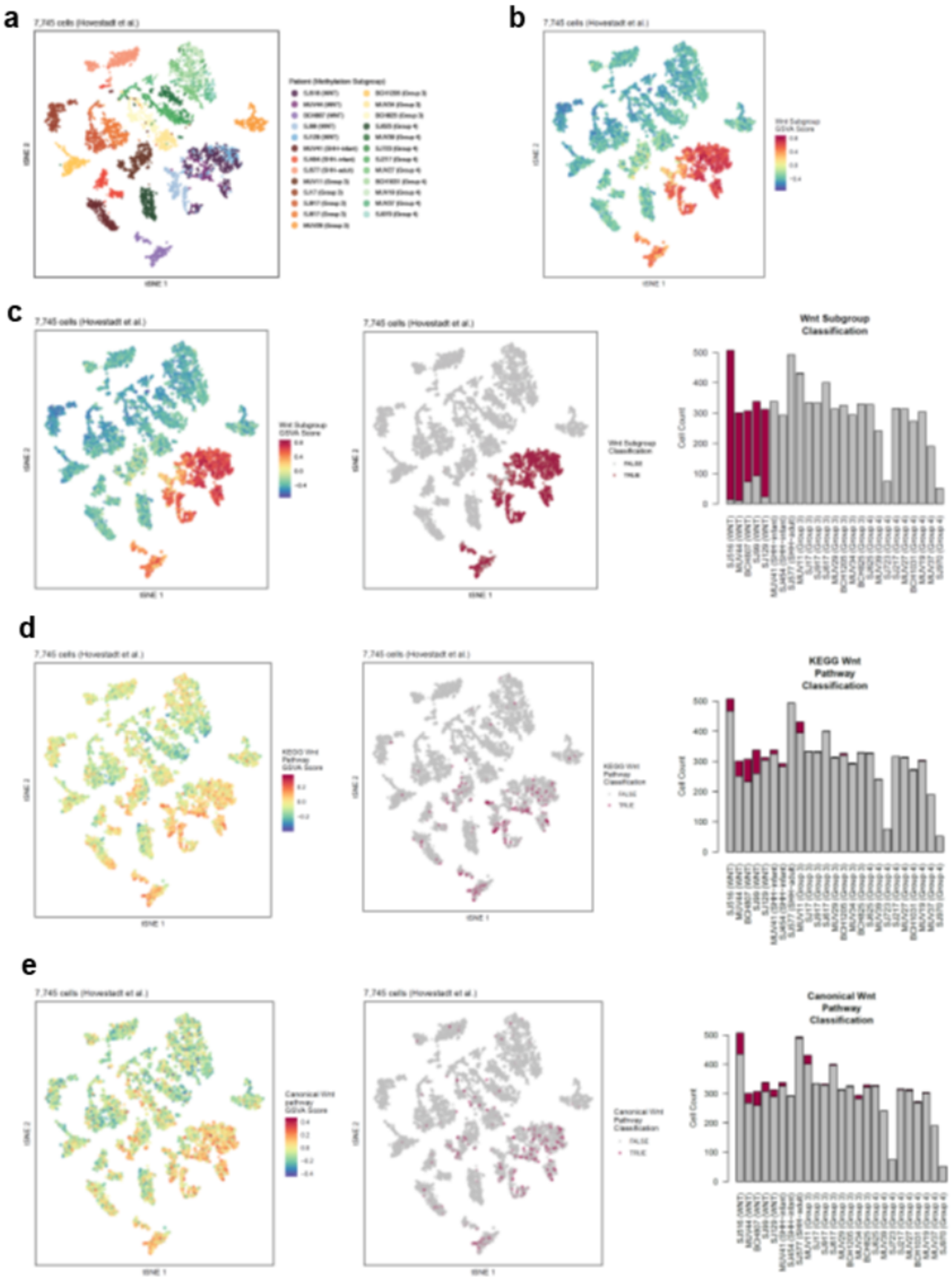


**Supplementary Figure 15: Single-cell RNA-seq data of 7,745 tumor cells demonstrates rare fraction of Wnt active cells in Shh, Group 3, and Group 4 MBs.** (a) Samples subgrouped according to methylation results from Hovestadt, *et al.*[27]. (b) All cells are malignant using annotations from Hovestadt, *et al.*[27]. (c) (left) tSNE colored by

Wnt subgroup GSVA score, (middle) tSNE colored by Wnt subgroup classification, (right) proportion Wnt subgroup + cells across samples. (d) (left) tSNE colored by KEGG Wnt pathway GSVA score, (middle) tSNE colored by KEGG Wnt pathway classification, (right) proportion KEGG Wnt pathway + cells across samples. (e) (left) tSNE colored by canonical Wnt pathway GSVA score, (middle) tSNE colored by canonical Wnt pathway classification, (right) proportion canonical Wnt pathway + cells across samples.

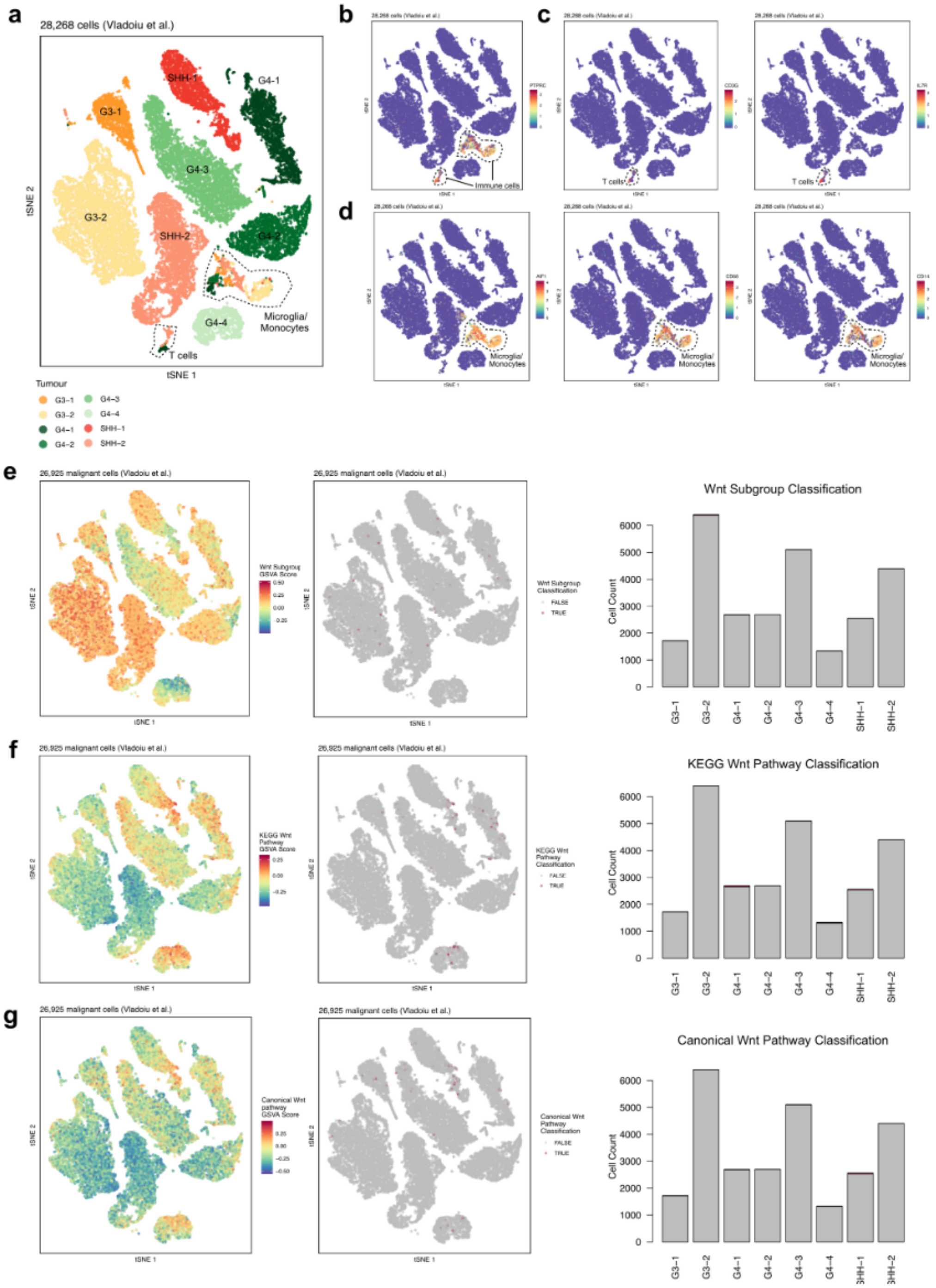


**Supplementary Figure 16: Single-cell RNA-seq data of 28,268 tumor cells demonstrates rare fraction of Wnt active cells in Group 3 and 4 MBs.** (a) tSNE of 28,268 cells from 8 MBs. Cells colored by sample ID from Vladoiu, *et al.*[28]. (b) Cells

colored by expression of pan-immune marker PTPRC (CD45). (c) Cells colored by expression of T cell markers CD3G (left) and IL7R (right). (d) Cells colored by expression of microglia/monocyte markers AIF1 (left), CD68 (middle), and CD14 (right). (e) Malignant cells colored by Wnt signaling GSVA score (left) and cell classification (middle). Bar plot depicting number of malignant cells classified as Wnt subgroup + per tumor (right). (f) Malignant cells colored by KEGG Wnt Pathway GSVA score (left) and cell classification (middle). Bar plot depicting number of malignant cells classified as KEGG Wnt Pathway + per tumor (right). (g) Malignant cells colored by canonical Wnt Pathway GSVA score (left) and cell classification (middle). Bar plot depicting number of malignant cells classified as canonical Wnt pathway + per tumor (right).

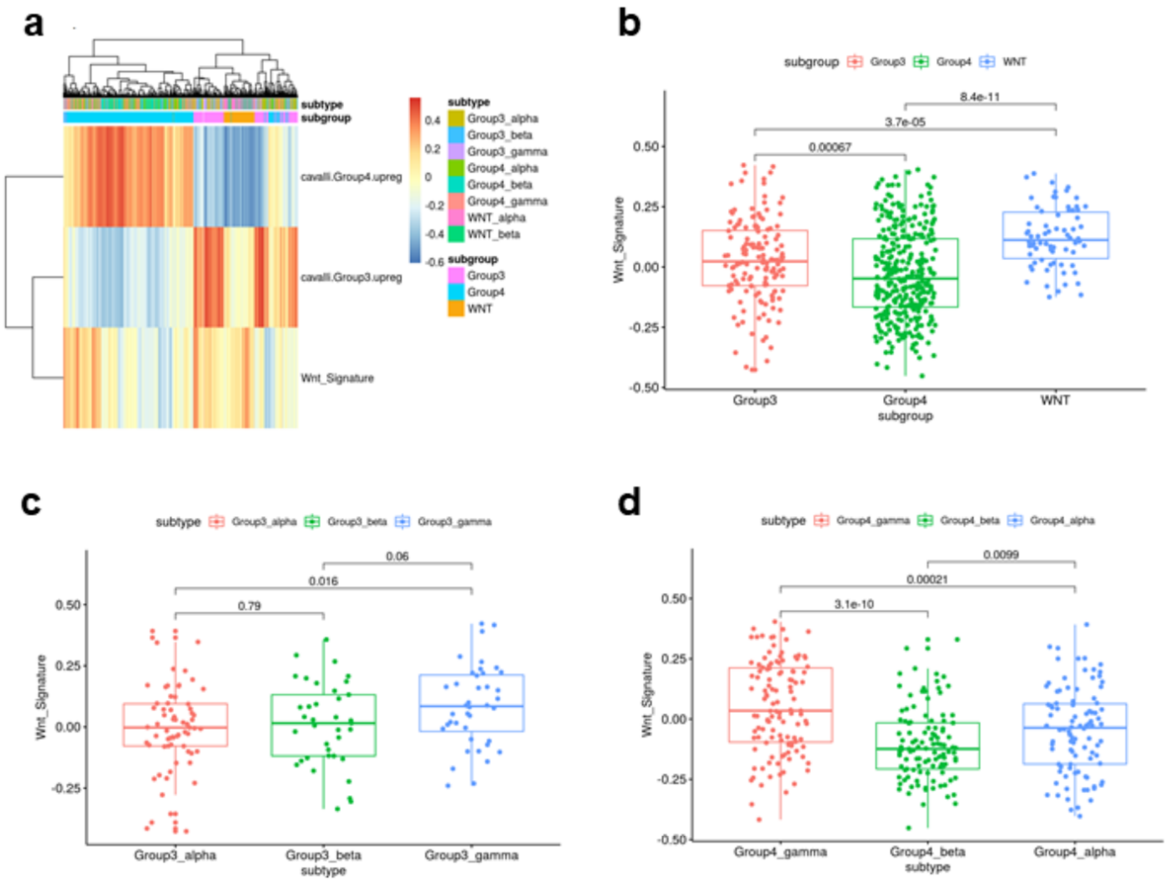


**Supplementary Figure 17: Subset of Group 3 and 4 MBs contain enhanced Wnt gene expression at bulk tumor level.** (a) GSVA scores were calculated for Wnt (n=170 patients), Group 3 (n=144 patients), Group 4 (n=326 patients), MBs from Cavalli *et al.*[10]. Heatmap of GSVA scores across Wnt, Group 3, Group 4 samples with heatmap cells colored by GSVA score, and ribbons indicating sample subtype and sample subgroup. Comparison of Broad Institute Wnt signature among (b) subgroups (Wnt, n=70 patients; Group 3, n=144 patients; Group 4, n=326 patients) (c) Group 3 subtypes (Group 3α, n=67 patients; Group 3ß, n=37 patients; Group 3γ, n=48 patients), and (d) Group 4 subtypes (Group 4α, n=98 patients; Group 4ß, n=109 patients; Group 4γ, n=119 patients). All tests were two-tailed. P-values for Wilcoxon rank-sum tests are shown. Boxplots are shown

with upper and lower whiskers defined as 1.5x IQR above and below median, respectively. The upper and lower lines of the box are defined as the 25th and 75th percentile, respectively, and the thick middle line of the box is defined as the median. Dots shown are simply a jitter plot of datapoints (not exclusive to outliers) to help visualize data distribution.

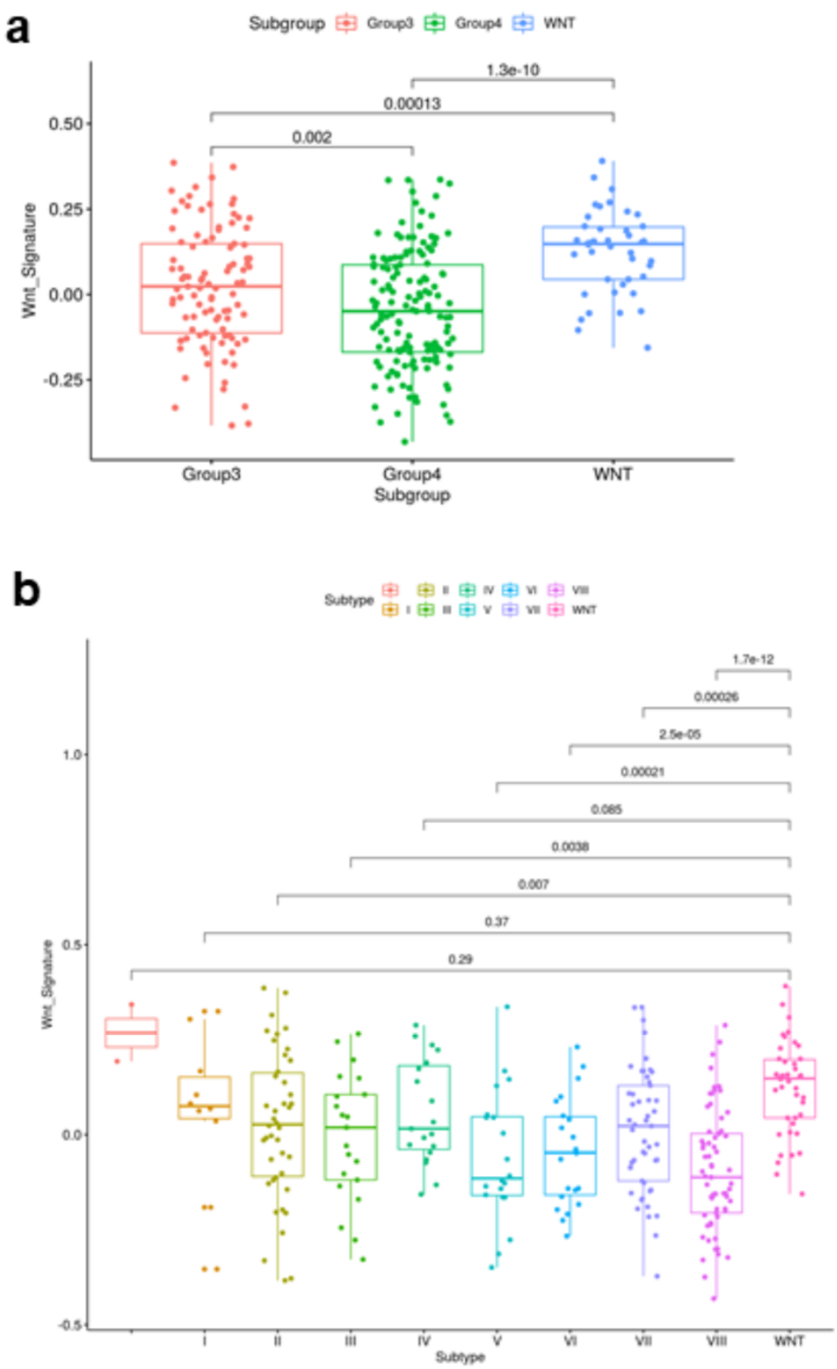


**Supplementary Figure 18: Methylation subtypes of Group 3 and 4 MBs contain variable Wnt expression.** (a) GSVA scores were calculated for Wnt (n=42 patients), Group 3 (n=101 patients), and Group 4 (n=149 patients) subgroup MBs from Northcott *et al.*[1] using the Broad Institute Wnt signature. Comparisons of Wnt signature were done

among (a) subgroups (Wnt, n=42 patients; Group 3, n=101 patients; Group 4, n=149 patients) and (b) between Group 3/Group 4 subtypes (unclassified (blank), n=2 patients; subtype I, n=10 patients; subtype II, n=42 patients; subtype III, n=21 patients; subtype IV, n=19 patients; subtype V, n=20 patients; subtype VI, n=22 patients; subtype VII, n=49 patients; subtype VIII, n=65 patients).  and the Wnt subgroup (n=42 patients). P-values for pairwise comparisons from two-sided t-test are shown. Boxplots are shown with upper and lower whiskers defined as 1.5x IQR above and below median, respectively. The upper and lower lines of the box are defined as the 25th and 75th percentile, respectively, and the thick middle line of the box is defined as the median. Dots shown are simply a jitter plot of datapoints (not exclusive to outliers) to help visualize data distribution.

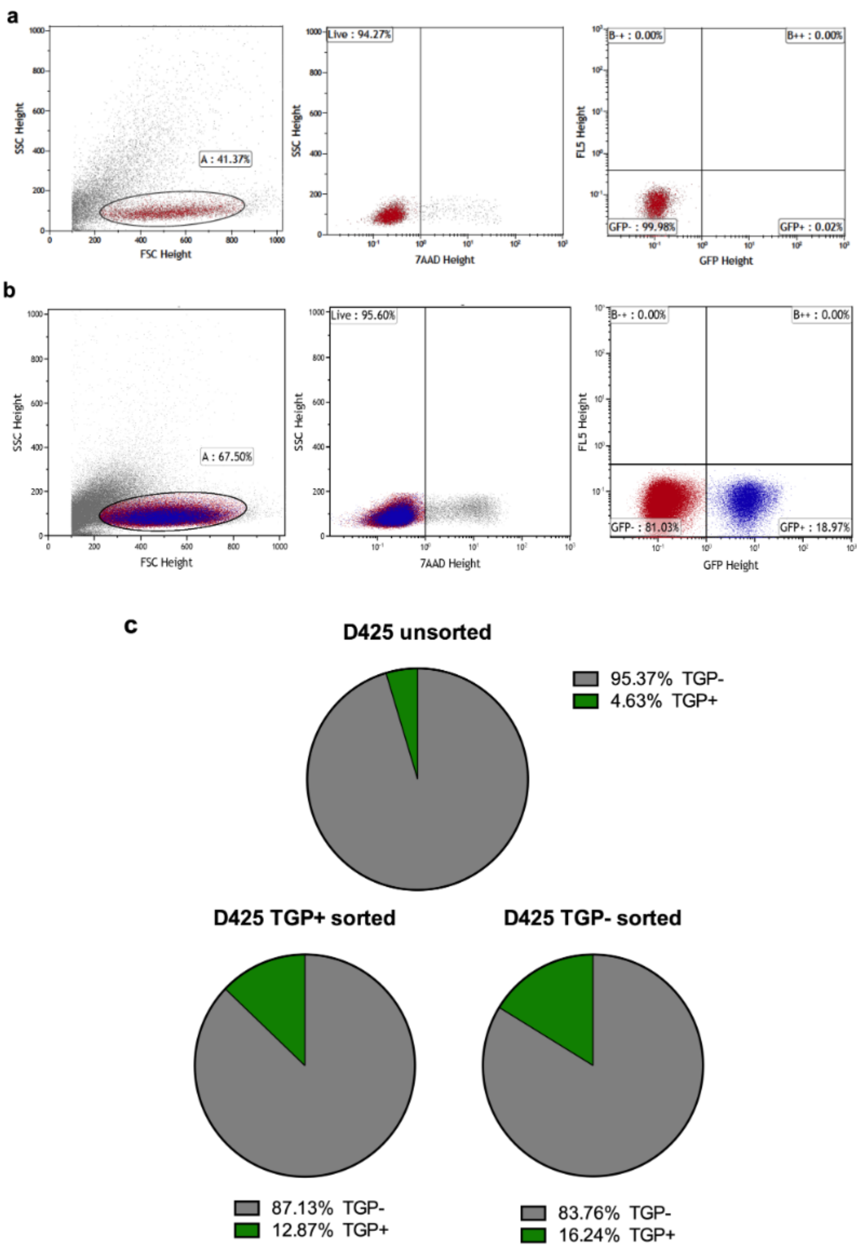


**Supplementary Figure 19: Wnt active (TGP+) and non-Wnt active (TGP-) show dynamic Wnt activity *in vitro*.** FSC-Height vs. SSC-Height is used as the initial gate to exclude debris. Viability gate is set using 7-AAD dye to exclude non-viable cells. Untransduced control is used to set the gate for expression of GFP, where gate is drawn

to distinguish between GFP-negative and GFP-positive populations. (a) Representative flow cytometric cell sorting plot for endogenous GFP+ (red) Wnt-active cells (TGP+) and (b) GFP- (blue) non-Wnt-active cells (TGP-). (c) TGP+ and TGP- D425 (Group 3) cells that were sorted and cultured separately show variation in GFP expression by flow cytometric analysis.

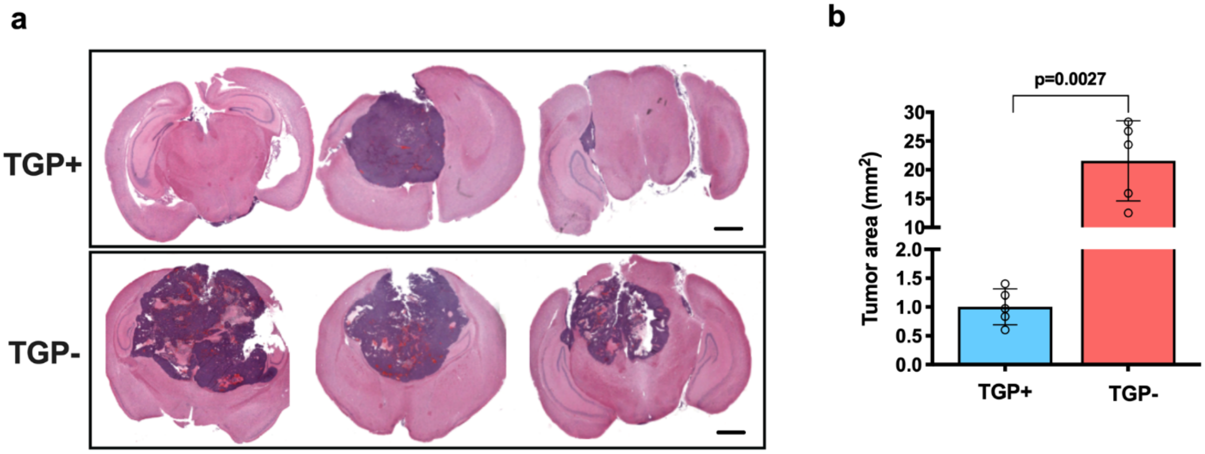


**Supplementary Figure 20: Wnt activation through ectopic mechanisms reduces overall tumor burden.** (a) Representative xenografts generated from TGP+ (n=6 mice) and TGP- (n=6 mice) cells from HD-MB03. (b) Xenografts (n=10 mice) generated from SU_MB002 TGP+ cells contain smaller tumor volumes when compared to control xenografts (n=10 mice) (p=0.0027). Error bars expressed as mean ± standard error (mean) using two-tailed, unpaired Student's t-test. Scale bar=5000 μm .

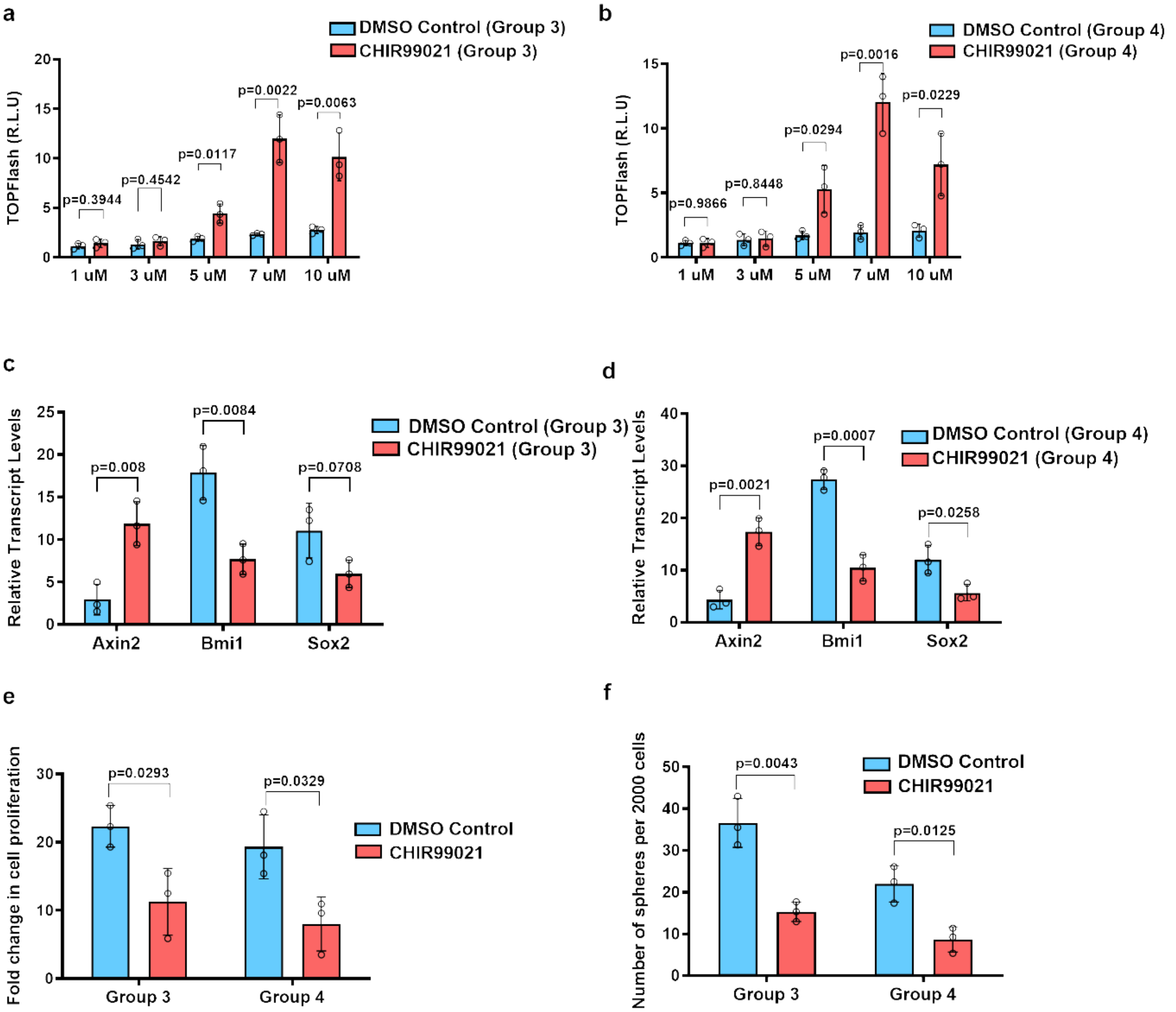


**Supplementary Figure 21: Small molecule (CHIR99021) Wnt activation impairs key stem cell properties in treatment-refractory MB lines.** (a) TCF Wnt reporter assay at various concentrations of CHIR99021 demonstrates optimal concentration for Wnt activation in Group 3 MBs (1μM: p=0.3944, 3μM: p=0.4542, 5μM: p=0.0117, 7μM: p=0.0022, 10 μM: p=0.0063) (n=3, independent experiments, 1 line per subgroup). (b) Similarly, TCF Wnt reporter assay at various concentrations of CHIR99021 demonstrates optimal concentration for Wnt activation in Group 4 MBs (1μM: p=0.9866, 3μM: p=0.8448,

5μM: p=0.0294, 7μM: p=0.0016, 10 μM: p=0.0229) (n=3, independent experiments, 1 line per subgroup). Differential *Axin2* (Group 3: p=0.008, Group 4: p=0.0021), *Bmi1* (Group 3: p=0.0084, Group 4: p=0.0007), and *Sox2* (Group 3: p=0.0708, Group 4: p=0.0258) transcript levels in CHIR99021-treated (c) Group 3 and (d) Group 4 MB lines (n=3, independent experiments per MB line, 1 line per subgroup, all samples normalized to *GAPDH*). Both, (e) proliferation (Group 3: p=0.0293, Group 4: p=0.0329) and (f) self-renewal (Group 3: p=0.0043, Group 4: p=0.0125) are impaired following CHIR99021 treatment in Group 3 and Group 4 MB lines (n=3, independent experiments per MB line, 1 line per subgroup). Error bars expressed as mean ± standard error (mean) using two-tailed, unpaired Student's t-test.

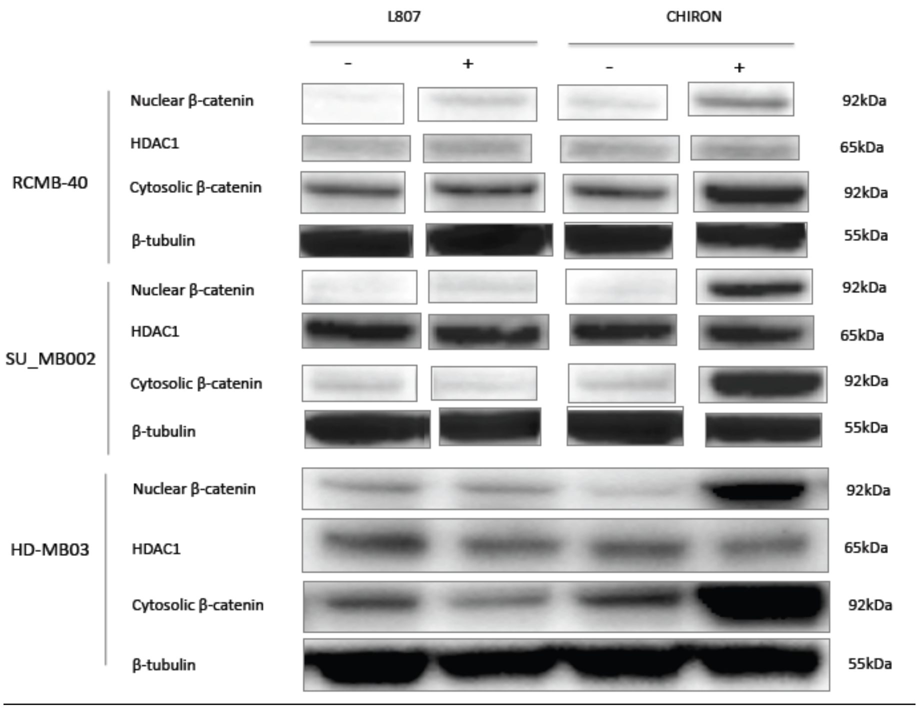


**Supplementary Figure 22: Wnt activation increases intracellular β-catenin levels in Group 3 MBs.** Both cytoplasmic and nuclear extracts from cells treated with CHIR99021 or L80Smts exhibit increased β-catenin levels (n=3 independent experiments for MB lines RCMB-40 and SU_MB002 and n=1 for HD-MB03).

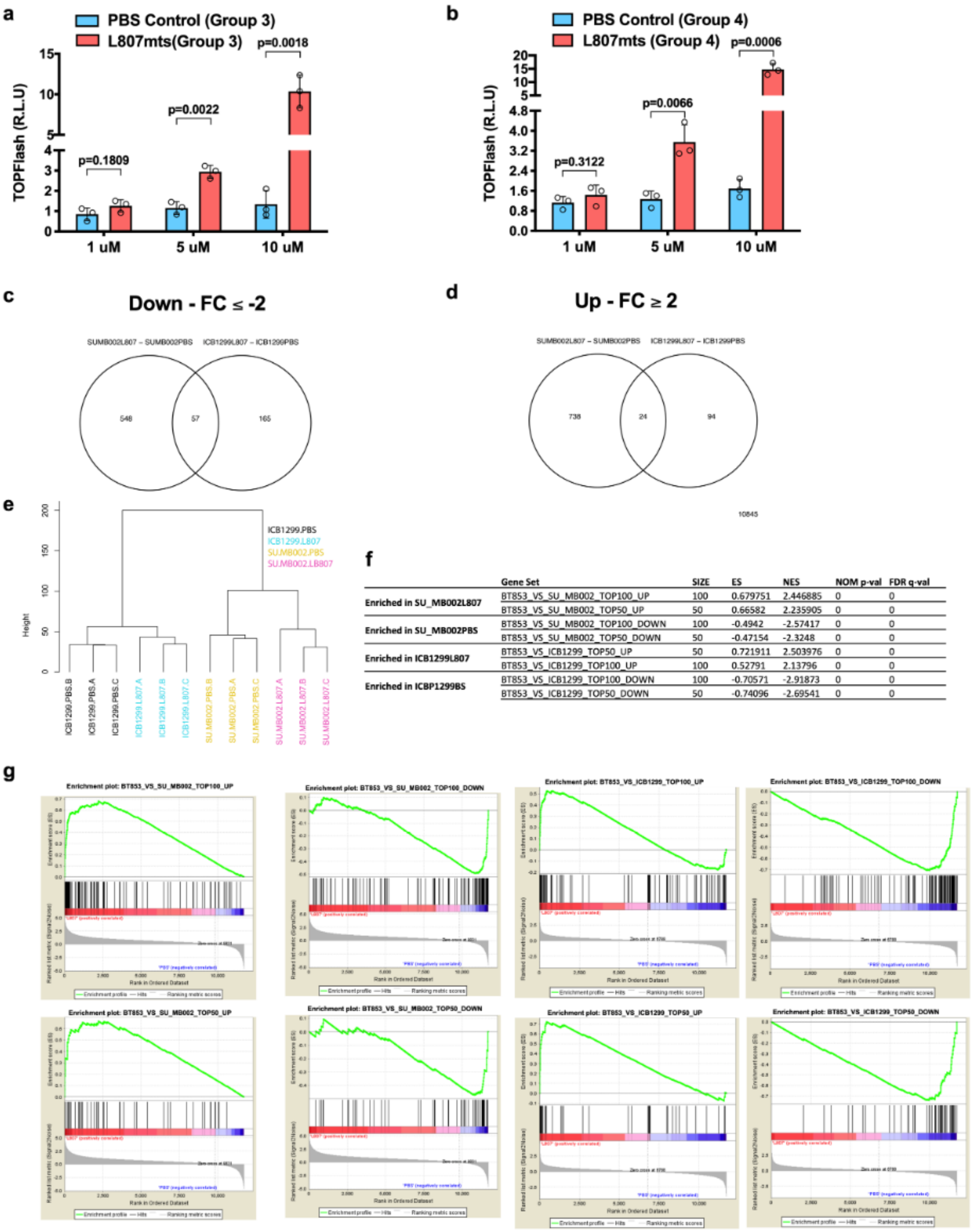


| | Gene Set | SIZE | ES | NES | NOM p-val | FDR q-val |
|---|---|---|---|---|---|---|
| Enriched in SU_MB002L807 | BT853_VS_SU_MB002_TOP100_UP | 100 | 0.679751 | 2.446885 | 0 | 0 |
| | BT853_VS_SU_MB002_TOP50_UP | 50 | 0.66582 | 2.235905 | 0 | 0 |
| Enriched in SU_MB002PBS | BT853_VS_SU_MB002_TOP100_DOWN | 100 | -0.4942 | -2.57417 | 0 | 0 |
| | BT853_VS_SU_MB002_TOP50_DOWN | 50 | -0.47154 | -2.3248 | 0 | 0 |
| Enriched in ICB1299L807 | BT853_VS_ICB1299_TOP50_UP | 50 | 0.721911 | 2.503976 | 0 | 0 |
| | BT853_VS_ICB1299_TOP100_UP | 100 | 0.52791 | 2.13796 | 0 | 0 |
| Enriched in ICBP1299BS | BT853_VS_ICB1299_TOP100_DOWN | 100 | -0.70571 | -2.91873 | 0 | 0 |
| | BT853_VS_ICB1299_TOP50_DOWN | 50 | -0.74096 | -2.69541 | 0 | 0 |

**Supplementary Figure 23: Wnt activation through substrate-competitive peptide Wnt agonist (L807mts) alters the transcriptional profile of Group 3 and 4 MBs and reduces their tumorigenic potential.** (a) TCF Wnt reporter assay at various concentrations of L807mts demonstrates optimal concentration for Wnt activation in Group 3 MBs (1μM: p=0.1809, 5μM: p=0.0022, 10 μM: p=0.0018) (n=3, independent

experiments, 1 line per subgroup). (b) Similarly, TCF Wnt reporter assay at various concentrations of L807mts demonstrates optimal concentration for Wnt activation in Group 4 MBs (1μM: $p=0.3122$, 5μM: $p=0.0066$, 10 μM: $p=0.0006$) ($n=3$, independent experiments, 1 line per subgroup). Venn diagrams of differentially (c) downregulated and (d) upregulated genes between L807mts- and PBS control-treated Group 3 and 4 MB lines ($n=3$, independent samples per MB line, 1 line per subgroup, FC=fold change) (e) Dendrogram of L807mts- and PBS control-treated Group 3 and 4 MB samples submitted for RNA-seq ($n=3$, independent samples per MB line). (f, g) GSEA shows evidence for activation of Wnt in L807mts treated Group 3 and 4 MB samples. Histology image scale bar = 5000 μm. Figures (a) and (b) contain error bars expressed as mean ± standard error (mean) using two-tailed, unpaired Student's t-test. Figure (f) is a result of the GSEA analysis (Kolmogorov Smirnov (K-S) test) and p-values corrected for multiple hypothesis testing (see FDR p-value).

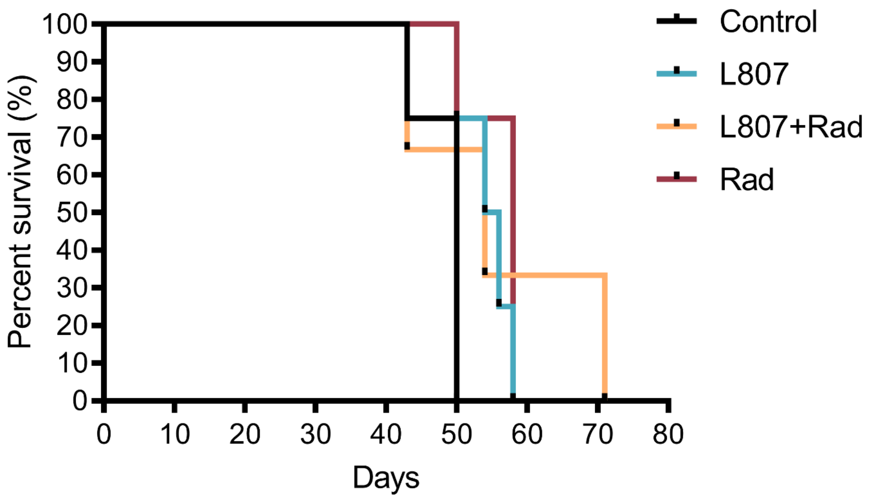


**Supplementary Figure 24: Group 3 MB xenografts treated with L807mts and radiation display trend for improved survival when compared to control xenografts.** SU_MB002 xenografts treated with L807mts prior to radiation had a survival advantage when compared to those treated with radiation alone (n=4 per group; median survival control: 50 days, L807mts: 55 days, L807mts+radiation: 54 days, radiation: 58 days; control vs. L807mts p=0.1095; control vs. L807mts+radiation p=0.2278; control vs. radiation p=0.0379). Figure was analyzed using log-rank (Mantel-Cox) test.

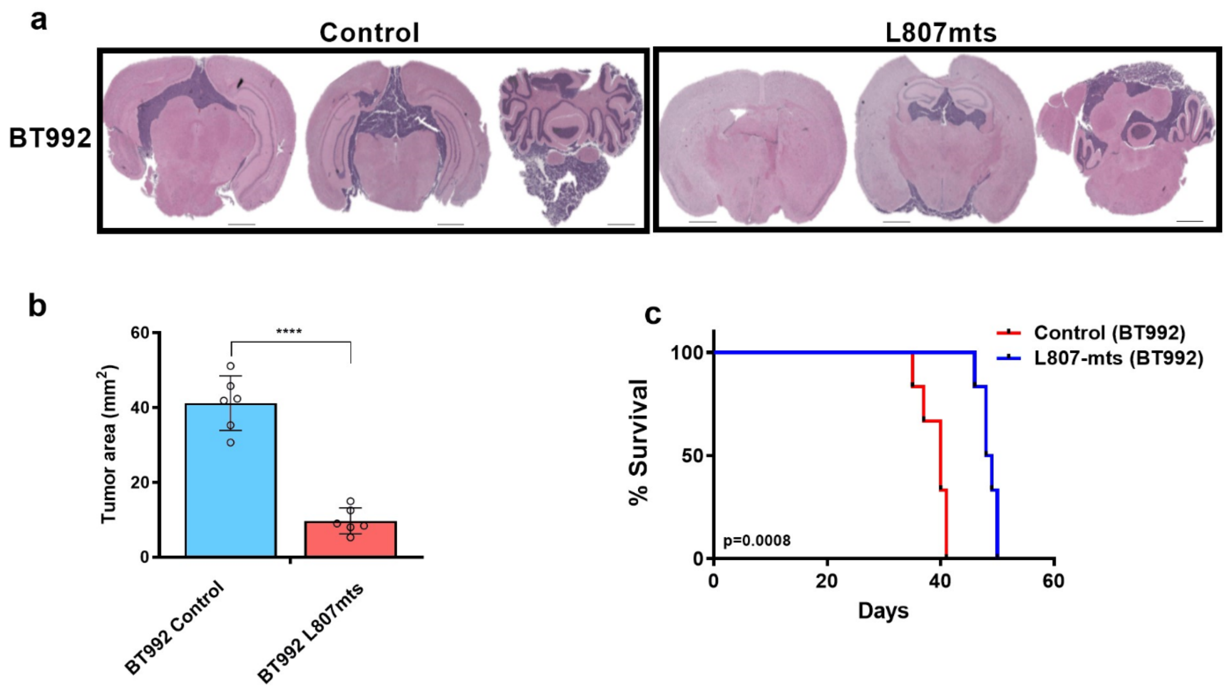


**Supplementary Figure 25: Wnt activation through Wnt agonist (L807mts) reduces the overall tumor burden of Shh MB xenografts.** (a) Representative histology images of xenografts generated from Shh MB (BT992) following L807mts treatment contain a reduction in overall tumor burden when compared to xenografts treated with PBS control (n=6 mice). (b) Shh MB (BT992) xenografts treated with L807mts (n=6 mice) contain a significant reduction in overall tumor volume (p=0.000026) and (c) increase in survival advantage (n=12 mice; median survival L807mts 48.5 days, PBS 40.0 days; p=0.0008) when compared to PBS control-treated xenografts. Scale bar: 5000 µm. Figure (b) contains error bars expressed as mean ± standard error (mean) using two-tailed, unpaired Student's t-test. Figure (c) was analyzed using log-rank (Mantel-Cox) test. **** p<0.0001.

a

## Blots used in Figure 1b

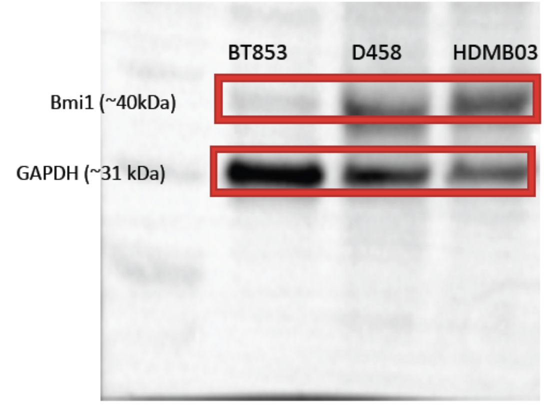


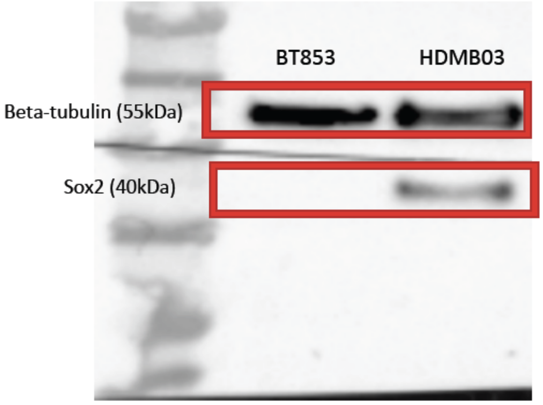


b

## Blots used in Sup Fig 22

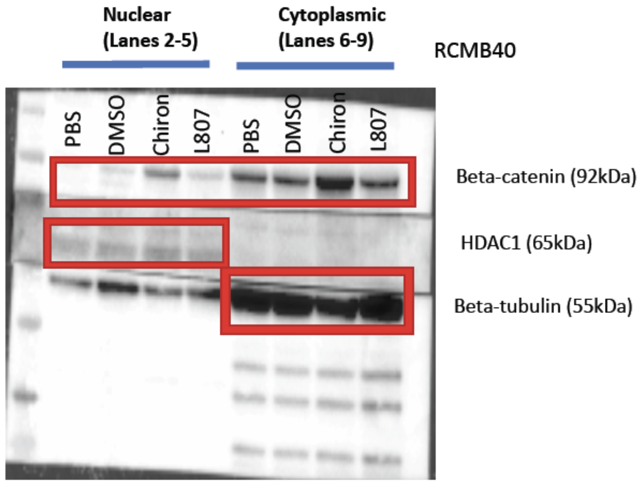


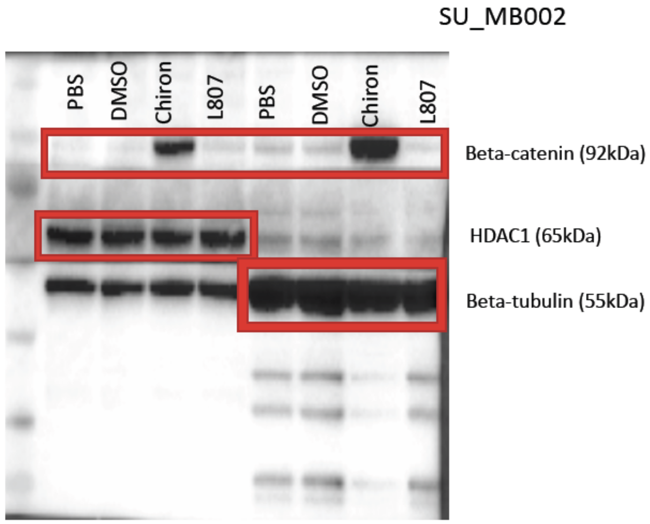


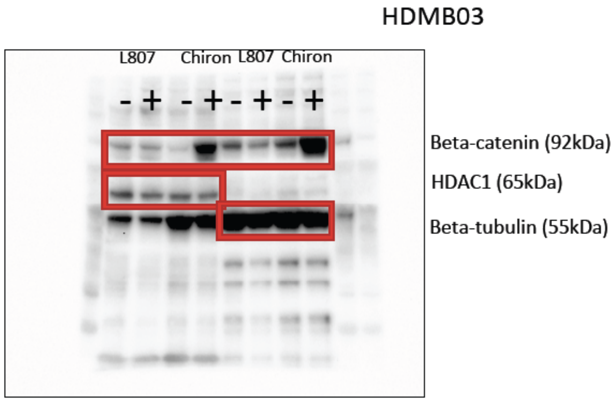

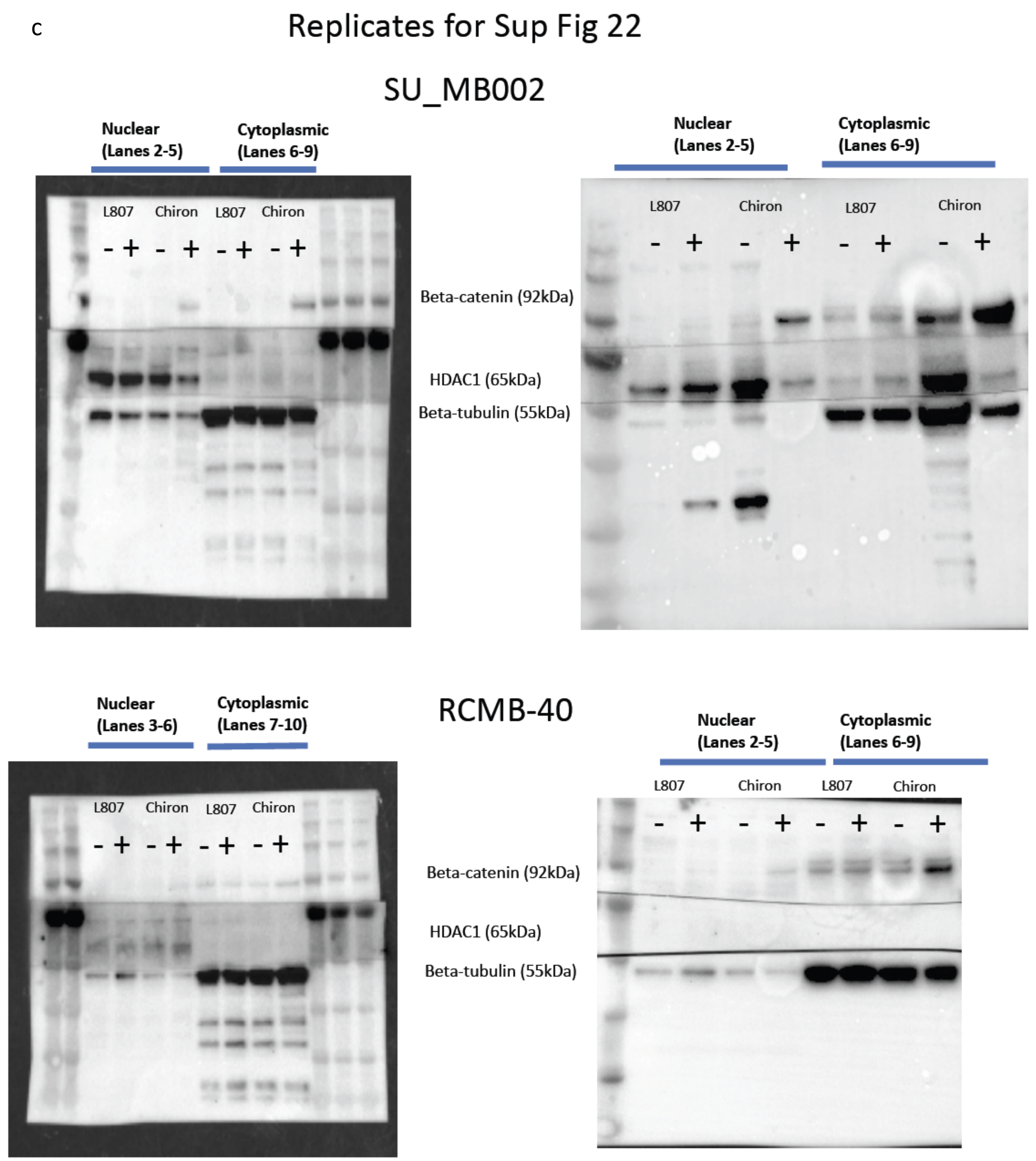


**Supplementary Figure 26: Uncropped and unprocessed scans of western blots.** Western blots presented in (a) Figure 1b and (b) Supplementary Figure 22 (c) Replicates for Supplementary Figure 22.

## Supplementary Tables

| Bmi1 (Glinsky signature) | | | | | | |
|---|---|---|---|---|---|---|
| Groups 3 and 4 | | | | | | |
| Using Index Group (High vs. Low) [Quantile 66] | | | | | | |
| Characteristic | Description | N | Hazard Ratio | (95% CI) | p-value | |
| Univariate analyses | | | | | | |
| **Index Group** | **High/Low** | **377** | **0.62** | **0.43-0.90** | **0.0113** | * |
| Age | <=3years/>3years | **373** | **2.05** | **1.20-3.49** | **0.00779** | ** |
| Metastatic status | M0/M+ | 343 | 1.32 | 0.90-1.94 | 0.153 | |
| **Subgroup** | **Group 3/Group4** | **377** | **0.55** | **0.38-0.79** | **0.00137** | ** |
| | | | | | | |
| | | | | | | |
| Multivariate analysis | p-value = 0.0004774 | | n=339 | | | |
| **Index Group** | **High/Low** | 339 | **0.65** | **0.44-0.96** | **0.0287** | * |
| Age | <=3years/>3years | | 1.45 | 0.82-2.58 | 0.20499 | |
| Metastatic status | M0/M+ | | 0.79 | 0.53-1.16 | 0.22704 | |
| **Subgroup** | **Group 3/Group4** | | **0.55** | **0.37-0.83** | **0.00421** | ** |

| Associations with Index (Log2 expression) | | | | |
|---|---|---|---|---|
| Characteristic | Statistic | N | Mean (SD) Index | p-value |
| Age | <=3years | 35 | 7.69 (0.32) | 0.94 |
| | >3years | 351 | 7.70 (0.28) | |
| Metastatic status | M0 | 214 | 7.67 (0.29) | 0.03339 |
| | M+ | 139 | 7.73 (0.28) | |
| Subgroup | Group 3 | 115 | 7.67 (0.35) | 0.3761 |
| | Group 4 | 275 | 7.71 (0.26) | |

**Supplementary Table 1:** Univariate and multivariate analyses of combined Groups 3 and 4 patients based on the *Bmi1* signature. Analysis performed using fitting Cox proportional hazards regression models. No multiple testing correction for univariate or multivariate testing.

| Sox2 signature | | | | | | |
|---|---|---|---|---|---|---|
| Groups 3 and 4 | | | | | | |
| Using Index Group (High vs. Low) [Quantile 66] | | | | | | |
| Characteristic | Description | N | HazardRatio | (95% CI) | p-value | |
| Univariate analyses | | | | | | |
| **Index Group** | **High/Low** | 377 | **0.59** | **0.41-0.85** | **0.00441** | ** |
| Age | <=3years/>3years | 373 | **2.05** | **1.21-3.49** | **0.007791** | ** |
| Metastatic status | M0/M+ | 343 | 1.32 | 0.90-1.94 | 0.153 | |
| **Subgroup** | **Group 3/Group4** | 377 | **0.55** | **0.38-0.79** | **0.00137** | ** |
| | | | | | | |
| | | | | | | |
| Multivariate analysis | p-value = 0.001193 | | n=339 | | | |
| **Index Group** | High/Low | 339 | 0.72 | 0.49-1.06 | 0.0924 | . |
| Age | <=3years/>3years | | 1.46 | 0.82-2.59 | 0.1994 | |
| Metastatic status | M0/M+ | | 0.77 | 0.52-1.14 | 0.1897 | |
| **Subgroup** | **Group 3/Group4** | | **0.59** | **0.39-0.89** | **0.0114** | * |

| Associations with Index (Log2 expression) | | | | |
|---|---|---|---|---|
| Characteristic | Statistic | N | Mean (SD) Index | p-value |
| Age | <=3years | 35 | 8.18 (0.08) | 0.34 |
| Characteristic | >3years | 351 | 8.17 (0.06) | |
| Metastatic status | M0 | 214 | 8.16 (0.07) | 0.03888 |
| | M+ | 139 | 8.18 (0.06) | |
| Subgroup | Group 3 | 115 | 8.18 (0.08) | 0.02943 |
| | Group 4 | 275 | 8.16 (0.06) | |

**Supplementary Table 2:** Univariate and multivariate analyses of combined Groups 3 and 4 patients based on the *Sox2* signature. Analysis performed using fitting Cox proportional hazards regression models. No multiple testing correction for univariate or multivariate testing.

| WNT signature | | | | | | |
|---|---|---|---|---|---|---|
| Group 3 | | | | | | |
| Using Index Group (High vs. Low) [Quantile 66] | | | | | | |
| Characteristic | Description | N | HazardRatio | (95% CI) | p-value | |
| Univariate analyses | | | | | | |
| **Index Group** | **High/Low** | 113 | **2.45** | **1.18-5.07** | **0.0158** | * |
| Age | <=3years/>3years | 113 | 1.39 | 0.70-2.73 | 0.3475 | |
| Metastatic status | M0/M+ | 106 | 1.63 | 0.90-2.95 | 0.106 | |
| | | | | | | |
| | | | | | | |
| | | | | | | |
| Multivariate analysis | p-value = 0.02746 | | n=105 | | | |
| **Index Group** | **High/Low** | 105 | **2.73** | **1.27-5.88** | **0.0103** | ** |
| Age | <=3years/>3years | | 1.02 | 0.49-2.13 | 0.9491 | |
| Metastatic status | M0/M+ | | 0.61 | 0.33-1.12 | 0.1116 | |

| Associations with Index (Log2 expression) | | | | |
|---|---|---|---|---|
| Characteristic | Statistic | N | Mean (SD) Index | p-value |
| Age | <=3years | 24 | 8.25 (0.09) | 0.22 |
| | >3years | 90 | 8.28 (0.14) | |
| Metastatic status | M0 | 65 | 8.26 (0.13) | 0.1736 |
| | M+ | 42 | 8.30 (0.14) | |

**Supplementary Table 3:** Univariate and multivariate analyses of Groups 3 patients based on the *Wnt* signature. Analysis performed using fitting Cox proportional hazards regression models. No multiple testing correction for univariate or multivariate testing.
