# Supplementary material for: Wnt activation as a therapeutic strategy in medulloblastoma
Source: Nat Commun. 2020 Aug 28;11:4323. doi: 10.1038/s41467-020-17953-4 (PMC7455709; doi:10.1038/s41467-020-17953-4)
Supplement: Supplementary file 2 — Description of Additional Supplementary Information [file 41467_2020_17953_MOESM2_ESM.pdf]

Description of Additional Supplementary Files

File Name: Supplementary Data 1

Description: Maximal fold changes for RNA-seq differential gene expression profile. 2 genes (March 1 and March 2) were represented by 2 transcripts each with maximal fold changes per gene provided in the table. Only genes with a fold change of at least 2 are presented. Differential expression was obtained by using "limma" package, p-values were adjusted for multiple testing with BH method with p-<0.05 considered significant. Pathway analysis was performed using the Reactome tool (https://reactome.org/PathwayBrowser/#TOOL=AT), and only terms yielding FDR < 0.05 were considered to be significant and were used for further examination.

File Name: Supplementary Data 2

Description: Differential pathway networks enrichment. Upregulated and downregulated pathways between Wnt, Group 3, and Group 4 MBs or L807mts- and PBS control-treated samples. Differential expression was obtained by using "limma" package, p-values were adjusted for multiple testing with BH method with p-<0.05 considered significant. Pathway analysis was performed using the Reactome tool (https://reactome.org/PathwayBrowser/#TOOL=AT), and only terms yielding FDR < 0.05 were considered to be significant and were used for further examination.
