# Supplementary material for: Wnt activation as a therapeutic strategy in medulloblastoma
Source: Nat Commun. 2020 Aug 28;11:4323. doi: 10.1038/s41467-020-17953-4 (PMC7455709; doi:10.1038/s41467-020-17953-4)
Supplement: Supplementary file 5 — Reporting Summary [file 41467_2020_17953_MOESM5_ESM.pdf]

nature research

Corresponding author(s): Dr. Sheila Singh

Last updated by author(s): Jun 12, 2020

Reporting Summary

Nature Research wishes to improve the reproducibility of the work that we publish. This form provides structure for consistency and transparency in reporting. For further information on Nature Research policies, see Authors & Referees and the Editorial Policy Checklist .

Statistics

For all statistical analyses, confirm that the following items are present in the figure legend, table legend, main text, or Methods section.

n/a	Confirmed	
☐	☒	The exact sample size (*n*) for each experimental group/condition, given as a discrete number and unit of measurement
☐	☒	A statement on whether measurements were taken from distinct samples or whether the same sample was measured repeatedly
☐	☒	The statistical test(s) used AND whether they are one- or two-sided<br>*Only common tests should be described solely by name; describe more complex techniques in the Methods section.*
☒	☐	A description of all covariates tested
☒	☐	A description of any assumptions or corrections, such as tests of normality and adjustment for multiple comparisons
☐	☒	A full description of the statistical parameters including central tendency (e.g. means) or other basic estimates (e.g. regression coefficient) AND variation (e.g. standard deviation) or associated estimates of uncertainty (e.g. confidence intervals)
☐	☒	For null hypothesis testing, the test statistic (e.g. *F*, *t*, *r*) with confidence intervals, effect sizes, degrees of freedom and *P* value noted<br>*Give P values as exact values whenever suitable.*
☒	☐	For Bayesian analysis, information on the choice of priors and Markov chain Monte Carlo settings
☒	☐	For hierarchical and complex designs, identification of the appropriate level for tests and full reporting of outcomes
☐	☒	Estimates of effect sizes (e.g. Cohen's *d*, Pearson's *r*), indicating how they were calculated

Our web collection on statistics for biologists contains articles on many of the points above.

Software and code

Policy information about availability of computer code

Data collection	No software was used for data collection
Data analysis	GraphPad Prism 5 (GraphPad Software Inc., La Jolla, California, USA)<br>R (https://www.r-project.org/) Version 2.3.4<br>Seurat (v2.3.4), limma version 3.38.3, GSVA version 1.30.0<br>Image J version 1.8.0_172<br>HISAT<br>rgl package<br>STATS package

For manuscripts utilizing custom algorithms or software that are central to the research but not yet described in published literature, software must be made available to editors/reviewers. We strongly encourage code deposition in a community repository (e.g. GitHub). See the Nature Research guidelines for submitting code & software for further information.

Data

Policy information about availability of data

All manuscripts must include a data availability statement. This statement should provide the following information, where applicable:

- Accession codes, unique identifiers, or web links for publicly available datasets
- A list of figures that have associated raw data
- A description of any restrictions on data availability

RNA seq data was downloaded from Cavalli et al., 2017 pediatric data set (http://gliovis.bioinfo.cnio.es) and Microarray array was provided by Northcott laboratory (Northcott et al., 2017)
ScRNA seq data was downloaded from GSE119926 and Vladoiu et al.,2019 (https://www.ncbi.nlm.nih.gov/pubmed/31043743).The RNA Seq data discussed in this publication have been deposited in NCBI's Gene Expression Omnibus (Edgar et al., 2002) and are accessible through GEO Series accession number GSE131473

(https://www.ncbi.nlm.nih.gov/geo/query/acc.cgi?acc=GSE131473).
The scRNA seq data have been deposited in CReSCENT (CRES-P22) https://pughlab.github.io/crescent-frontend/#section-4 and will be available upon request (will be made publically available soon) and processed data is on uploaded on BROAD institute site: https://singlecell.broadinstitute.org/single_cell/study/SCP840.

Field-specific reporting

Please select the one below that is the best fit for your research. If you are not sure, read the appropriate sections before making your selection.

- [x] Life sciences
- [ ] Behavioural & social sciences
- [ ] Ecological, evolutionary & environmental sciences

For a reference copy of the document with all sections, see nature.com/documents/nr-reporting-summary-flat.pdf

Life sciences study design

All studies must disclose on these points even when the disclosure is negative.

Sample size	In vivo sample sizes were chosen for in vivo studies based on prior studies, which validated the minimum number of mice to determine a significant difference. This was determined at a minimum of 5 mice per treatment arm.<br><br>In vitro sample sizes were partly limited based on number of available primary cell lines and replicates were performed at least 3 times independently for each available cell line.<br><br>All experiments were replicated and performed independently for a minimum of 3 times. All attempts at replication were successful and provide an overall reflection of each individual experiment's findings.
Data exclusions	No data were excluded.
Replication	All attempts at data replication were taken to confirm previous results.
Randomization	For L807mts treatment, each litter of NOD-SCID mice were randomized (in order of transplantation to control and treatment group. Otherwise, samples were allocated randomly to experimental groups.
Blinding	Investigators were blinded to group allocation. Macroscopic and/or microscopic phenotypes prevented blinding during data collection and analysis.

Reporting for specific materials, systems and methods

We require information from authors about some types of materials, experimental systems and methods used in many studies. Here, indicate whether each material, system or method listed is relevant to your study. If you are not sure if a list item applies to your research, read the appropriate section before selecting a response.

Materials & experimental systems

n/a	Involved in the study	
☐	☒	Antibodies
☐	☒	Eukaryotic cell lines
☒	☐	Palaeontology
☐	☒	Animals and other organisms
☐	☒	Human research participants
☒	☐	Clinical data

Methods

n/a	Involved in the study	
☒	☐	ChIP-seq
☐	☒	Flow cytometry
☒	☐	MRI-based neuroimaging

Antibodies

Antibodies used	Western blotting:<br>Mouse Anti human Bmi1 (1:1000; Millipore #051321, clone AF27)<br>Mouse Anti-human Sox2 (1:2,000; BD Biosciences #561469;Clone O30-678)<br>Rabbit antihuman β-catenin (1:500; Cell Signaling #9581)<br>Rabbit anti-human β-tubulin (1:50000; Abcam #ab6046).<br>Rabbit Anti-human HDAC1 (1:200 Millipore #06-720)<br>Horseradish peroxidase conjugated goat anti-mouse IgG (Bio-Rad) or goat anti-rabbit IgG (Sigma).<br><br>Flow analysis:<br>Recombinant Antibody: Anti-human Bmi1; PE conjugated (1:10 Miltenyi, #130-106-736)

	Mouse Anti-human Sox2 V450, (1:20 BD Biosciences # 561610; Clone O30-678)<br><br>Immunoflourescence (Sox2)<br>Rabbit anti-human polyclonal Sox2 antibody (1:100, Abcam 97959)<br>Alexa Flour 488 goat anti-rabbit IgG (1:200, Life Technologies)<br><br>Immunohistochemistry (GFP):<br>Rabbit polyclonal GFP primary antibody (Abcam; ab6556; 1:1000)<br>Donkey anti-rabbit biotin secondary antibody (Jackson ImmunoResearch Laboratories; 711-065-152; 1:1000)
Validation	Western antibodies:<br>Mouse Anti human Bmi1 - Validated by the manufacturer using MEF1 cell lysate, Our lab validated by using U2OS cell lysate as a positive control<br>Mouse Anti-human Sox2 -Validated by the manufacturer using Neural stem cells<br>Rabbit antihuman β-catenin- Validated by the manufacturer using total cell extracts from 293 and NIH/3T3 cells<br>Rabbit anti-human β-tubulin- Validated by the manufacturer using HeLa Cell lysate; A431 Cell lysate; MCF7 Cell lysate; with and without beta tubulin peptide.<br>Rabbit Anti-human HDAC1 -Validated by the manufacturer using positive control NIH/3T3 cell extract<br><br>Flow analysis:<br>Recombinant Antibody: Anti-human Bmi1; PE conjugated - Validated by the manufacturer using HeLa Cells<br>Mouse Anti-human Sox2 V450- Validated by the manufacturer using Embryonic stem cells<br><br>Immunoflourescence (Sox2)<br>Rabbit anti-human polyclonal Sox2 antibody has been validated by the manufacturer on E13 mouse spinal cord sections.<br><br>Immunohistochemistry (GFP)<br>ab6556 has been referenced in 836 publications.

Eukaryotic cell lines

Policy information about cell lines

Cell line source(s)	Primary human pediatric MBs, BT853 and BT992 were obtained from consenting patients and families as approved by the Hamilton Health Sciences/McMaster Health Sciences Research Ethics Board. Additional primary human pediatric MB cultures were obtained from collaborators as kind gifts. SU_MB002, RCMB-40, and ICB1299 were received from Dr. Yoon-Jae Cho, Dr. Robert Wechsler-Reya, and Dr. Silvia Marino, respectively. HD-MB03 was obtained from Dr.Till Mildy. Daoy, D425, D458,Med8A are commercially available cell lines from ATCC (https://www.atcc.org/en.aspx).
Authentication	STR profiling
Mycoplasma contamination	All cell lines were tested negative for mycoplasma
Commonly misidentified lines<br>(See ICLAC register)	No commonly misidentified cell lines were used

Animals and other organisms

Policy information about studies involving animals; ARRIVE guidelines recommended for reporting animal research

Laboratory animals	Male and female laboratory NOD-SCID mice of at least 12 weeks of age were used. Mice were maintained in the animal facilities at the McMaster University Stem Cell Unit (SCU) within the Animal Facility (CAF). They were maintained in a pathogen-free, temperature-controlled, 12h light and dark cycle environment and were fed ad libitum
Wild animals	No wild animals were used in this study
Field-collected samples	No field- collected samples were used in this study
Ethics oversight	All in vivo experiments were performed in accordance to the McMaster University Animal Research Ethics Board (AREB) approved protocols national guidelines and regulations.

Note that full information on the approval of the study protocol must also be provided in the manuscript.

Human research participants

Policy information about studies involving human research participants

Population characteristics	NA
Recruitment	NA
Ethics oversight	NA

Note that full information on the approval of the study protocol must also be provided in the manuscript.

Flow Cytometry

Plots

Confirm that:

- [x] The axis labels state the marker and fluorochrome used (e.g. CD4-FITC).
- [x] The axis scales are clearly visible. Include numbers along axes only for bottom left plot of group (a 'group' is an analysis of identical markers).
- [x] All plots are contour plots with outliers or pseudocolor plots.
- [x] A numerical value for number of cells or percentage (with statistics) is provided.

Methodology

Sample preparation	Adherent cells were dissociated with TryplE and suspended in PBS+0.5M EDTA to achieve a single cell suspension with subsequent filtration to remove clumps.
Instrument	MoFlo XDP cell sorter (Beckman Coulter)
Software	Kaluza 2.0 from Beckman Coulter
Cell population abundance	A small fraction of the sorted population was re-run through the MoFlo XDP to check for purity using the same gating strategy. Purity is determined by the percentage of cells that falls under the same gating used for sort. If purity is greater than 95%, sample is considered pure.
Gating strategy	For Bmi/Sox2 Flow anlaysis: (a)FSC-Height vs. SSC-Height is used as the initial gate to exclude debris (b) Viability gate is set using Live/Dead Fixable Near-IR stain to exclude non-viable cells (c) Unstained control is used to set the gate for expression of Bmi1, CD133 and Sox2, where gate is drawn to exclude baseline expression of fluorophores.<br>For GFP sorts: FSC-Height vs. SSC-Height is used as the initial gate to exclude debris. Viability gate is set using 7-AAD dye to exclude non-viable cells. Untransduced control is used to set the gate for expression of GFP, where gate is drawn to distinguish between GFP-negative and GFP-positive populations.

- [x] Tick this box to confirm that a figure exemplifying the gating strategy is provided in the Supplementary Information.
